# Metabolic Reprogramming during Purine Stress in the Protozoan Pathogen *Leishmania donovani*

**DOI:** 10.1371/journal.ppat.1003938

**Published:** 2014-02-27

**Authors:** Jessica L. Martin, Phillip A. Yates, Radika Soysa, Joshua F. Alfaro, Feng Yang, Kristin E. Burnum-Johnson, Vladislav A. Petyuk, Karl K. Weitz, David G. Camp, Richard D. Smith, Phillip A. Wilmarth, Larry L. David, Gowthaman Ramasamy, Peter J. Myler, Nicola S. Carter

**Affiliations:** 1 Department of Biochemistry & Molecular Biology, Oregon Health & Science University, Portland, Oregon, United States of America; 2 Division of Biological Sciences, Pacific Northwest National Laboratory, Richland, Washington, United States of America; 3 Proteomics Shared Resource Core, Oregon Health & Science University, Portland, Oregon, United States of America; 4 Seattle Biomedical Research Institute, Seattle, Washington, United States of America; 5 Department of Global Health and Department of Biomedical Informatics & Medical Education, University of Washington, Seattle, Washington, United States of America; University of Michigan, United States of America

## Abstract

The ability of *Leishmania* to survive in their insect or mammalian host is dependent upon an ability to sense and adapt to changes in the microenvironment. However, little is known about the molecular mechanisms underlying the parasite response to environmental changes, such as nutrient availability. To elucidate nutrient stress response pathways in *Leishmania donovani*, we have used purine starvation as the paradigm. The salvage of purines from the host milieu is obligatory for parasite replication; nevertheless, purine-starved parasites can persist in culture without supplementary purine for over three months, indicating that the response to purine starvation is robust and engenders parasite survival under conditions of extreme scarcity. To understand metabolic reprogramming during purine starvation we have employed global approaches. Whole proteome comparisons between purine-starved and purine-replete parasites over a 6–48 h span have revealed a temporal and coordinated response to purine starvation. Purine transporters and enzymes involved in acquisition at the cell surface are upregulated within a few hours of purine removal from the media, while other key purine salvage components are upregulated later in the time-course and more modestly. After 48 h, the proteome of purine-starved parasites is extensively remodeled and adaptations to purine stress appear tailored to deal with both purine deprivation and general stress. To probe the molecular mechanisms affecting proteome remodeling in response to purine starvation, comparative RNA-seq analyses, qRT-PCR, and luciferase reporter assays were performed on purine-starved *versus* purine-replete parasites. While the regulation of a minority of proteins tracked with changes at the mRNA level, for many regulated proteins it appears that proteome remodeling during purine stress occurs primarily *via* translational and/or post-translational mechanisms.

## Author Summary

*Leishmania*, the cause of a deadly spectrum of diseases in humans, surmounts a number of environmental challenges, including changes in the availability of salvageable nutrients, to successfully colonize its host. Adaptation to environmental stress is clearly of significance in parasite biology, but the underlying mechanisms are not well understood. To simulate the response to periodic nutrient scarcity *in vivo*, we have induced purine starvation *in vitro*. Purines are essential for growth and viability, and serve as the major energy currency of cells. *Leishmania* cannot synthesize purines and must salvage them from the surroundings. Extracellular purine depletion in culture induces a robust survival response in *Leishmania*, whereby growth arrests, but parasites persist for months. To profile the events that enable endurance of purine starvation, we used shotgun proteomics. Our data suggest that purine starvation induces extensive proteome remodeling, tailored to enhance purine capture and recycling, reduce energy expenditures, and maintain viability of the metabolically active, non-dividing population. Through global and targeted approaches, we reveal that proteome remodeling is multifaceted, and occurs through an array of responses at the mRNA, translational, and post-translational level. Our data provide one of the most inclusive views of adaptation to microenvironmental stress in *Leishmania*.

## Introduction

*Leishmania* are protozoan parasites that are a significant human health burden, afflicting approximately 12 million people in 88 countries worldwide [1]. These parasites cause a spectrum of diseases in humans ranging from cutaneous ulcerative lesions that can be localized or diffuse; disfiguring mucocutaneous lesions that manifest in the nose, mouth, and throat cavities; to fatal hepato- or splenomegaly arising from a visceralizing form of the disease [1]. Due to the lack of an effective vaccine, management of leishmaniasis is predicated on just a few drugs, most of which exhibit toxic side effects and are costly and burdensome to administer, putting them beyond the reach of many of the affected countries. Of particular concern is the high level of resistance currently observed to the drug Pentostam, a mainstay of leishmaniasis treatment, especially in regions endemic for *Leishmania donovani*, the causative agent of deadly visceral leishmaniasis [2], [3]. Thus, there is a compelling need for better therapeutic approaches for combating leishmaniasis in humans.

One long-standing approach to defining new pathways and targets for drug design has been to identify parasite pathways that are both different from their host and vital for parasite viability [4]–[6]. An ability to adapt to host nutritional and physiological changes is a key feature of parasitism and ensures parasite survival, even under less than optimal conditions. *Leishmania*, in particular, overcome dramatic physiological alterations in their host milieu as they transition between extracellular promastigotes in the ambient, neutral environment inside of the sandfly to intracellular amastigotes that reside in the acidic phagolysosome of mammalian macrophages at 37°C [7]. It is likely that salvageable nutrients that are essential for parasite viability also fluctuate throughout the *Leishmania* lifecycle, since these parasites have evolved robust mechanisms to deal with periods of nutrient paucity [7]–[13]. Indeed, nutrient depletion, at least *in vitro*, can be used as a trigger for metacyclogenesis in *Leishmania* [14]–[16], as well as in the closely related *Trypanosoma cruzi* [17], [18], implying that nutrient stress provokes substantial changes to the parasite proteome. Therefore, these parasites must sense and adapt to changing extracellular and intracellular conditions as they colonize the different microenvironments of each host.

Despite their significance for parasite survival, little is known about the molecular mechanisms used by these parasites to respond to environmental changes, such as nutrient availability [19], [20]. Thus, to elucidate nutrient stress response pathways in *L. donovani*, we have used purine starvation as the paradigm. The salvage of purines from the host milieu is an obligatory process that impacts both cell viability and growth [21]. *Leishmania*, like all parasitic protozoa characterized to date, are auxotrophic for purines and have evolved a unique set of purine transporters and salvage enzymes to scavenge these essential nutrients from their host [21]–[23]. Because purine acquisition in *Leishmania* is an indispensable nutritional process, and the pathway is considered an attractive target for therapeutic exploration, the components of purine salvage have been extensively characterized at the molecular, biochemical, and, in some cases, at the structural level [21], [23]–[27]. However, the regulation of this pathway in response to changes in the extracellular purine milieu is poorly understood. Earlier studies in *Leishmania* and related parasites have revealed a marked augmentation in cell surface activities corresponding to 3′-nucleotidase/nuclease (**3′NT/NU**) and membrane-bound acid phosphatase in response to purine starvation [13], [28]–[32], and studies from our own laboratory [8], [33], as well as others [12], [34], [35], have shown augmentation of nucleoside and nucleobase transport activities and proteins. Purine starvation is easily induced *in vitro* by the withdrawal of purines from the growth medium, and we have previously developed conditions where the response to purine stress is both robust and readily tractable [8]. Our preliminary studies have shown that removal of purines from the extracellular milieu provokes striking morphological and metabolic changes [8]. Purine-starved parasites arrest growth after one division in G_1_/G_0_ phase of the cell cycle [8], and we have shown that they can persist in culture without the provision of purine for more than 3 months—during which,growth arrest is reversible by the addition of exogenous purine. Together, these observations suggest that the response to purine starvation is tailored for parasite survival even under extreme scarcity and that purine starvation in *Leishmania* is an ideal model for dissecting the response to nutrient stress.

The molecular mechanisms that lead to the upregulation of cell surface purine enzymes and transporters during purine stress are likely complex. Thus, as a first step towards uncovering the multi-faceted changes involved in adaptation to purine starvation, we have used global approaches. Here we describe an extensive comparison of the proteomes of purine-starved and purine-replete *L. donovani* over a 6–48 h window. These analyses have revealed that there is a temporal component to purine starvation, with earlier proteome changes tailored to counteract the scarcity of purine in the extracellular milieu and later proteome changes reflective of general responses to cellular stress that might accompany the reversible exit of the cells from the cell cycle. These later changes involve an extensive remodeling of the cellular proteome and likely contribute to the prolonged viability of these cells while under purine stress. Since *Leishmania* exhibit an unusual mechanism of gene regulation, whereby the majority of the leishmanial genome is constitutively transcribed and changes in protein abundance are directed by post-transcriptional mechanisms [36], [37], we have profiled post-transcriptional changes in mRNA stability by Whole Transcriptome Shotgun Sequencing or RNA-seq [38]–[45] to dissect the molecular mechanisms underlying proteome remodeling during purine stress. These analyses suggest that the post-transcriptional mechanisms that lead to proteome remodeling are complex and likely involve a diverse array of responses including changes in mRNA abundance, translational efficiency, as well as changes in the post-translational stability of proteins.

## Results

### Temporal Changes in the Leishmanial Proteome during Purine Restriction

**Figure 1:**
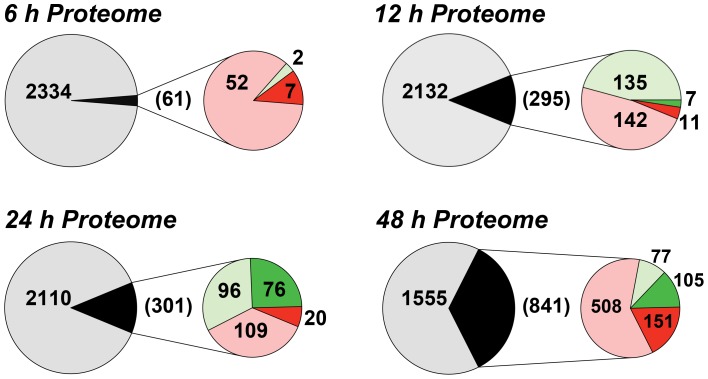
Summary of the proteome changes accompanying purine starvation. The proteomes of cells incubated in either purine-replete or in purine-deplete media were compared at 6, 12, 24, and 48 h post purine supplement removal. Large circles and the numbers within represent the total number of proteins that could be assigned between purine-replete and purine-deplete cells at each time point. Small circles and numbers in parentheses refer to the number of proteins significantly changed (p-value <0.05) at each time point between purine-replete and purine-deplete cells. Light red and light green fractions and corresponding numbers indicate proteins upregulated or downregulated, respectively, by less than 2-fold, dark red and dark green portions and corresponding numbers indicate proteins upregulated or downregulated, respectively, by 2-fold or more.

We have previously shown that the removal of extracellular purines leads to morphological changes in *L. donovani* promastigotes that manifest by 24 h post-purine removal from the growth medium [8]. Parasites starved for purines also cease growth and accumulate in G_1_/G_0_ phase of the cell cycle [8]. Accompanying these morphological and growth changes, *L. donovani* promastigotes also upregulate certain purine transport and salvage enzyme activities, as well as their corresponding proteins [8], [12]. To assess the additional effects of extracellular purine depletion upon the leishmanial proteome, we starved *L. donovani* promastigotes of purines over a 48 h time period and compared the proteome of these parasites with cells grown with an extracellular purine source at 6, 12, 24, and 48 h. Note that *Leishmania* are capable of growth in any of the naturally occurring purine nucleobases or nucleosides [21], but for these experiments 100 µM hypoxanthine was included as the sole purine supplement in the growth medium. Comparison of the proteomes of purine-starved and purine-replete parasites at each of these time points was performed by the label-free and ultra-sensitive proteomic accurate mass and time (**AMT**) tag method [46]–[48]. By AMT tag analysis, a total of 24,283 distinct peptides were identified corresponding to ∼4109 proteins, of which 2661 proteins were identified with ≥2 peptides (Table S1). (Note that the entire liquid chromatography-tandem mass spectrometry (**LC-MS/MS**) dataset for these analyses can be accessed at http://omics.pnl.gov/view/publication_1086.html). Given that the reference *Leishmania infantum* genome [49], [50], used in the generation of the theoretical peptide fragmentation library for these studies, comprises 8381 annotated protein coding sequences, this equated to a coverage of ∼49%. Of the ∼2500 proteins identified at each time point, ∼2.6, 12.2, 12.5, and 35.1% were observed to be significantly different in abundance (p-value ≤0.05) at the 6, 12, 24, and 48 h time points, respectively (Fig. 1 and Table S1). The median abundance change at 6, 12, 24 and 48 h for upregulated proteins was 1.49-, 1.36-, 1.58-, and 1.64-fold, respectively, and 0.69-, 0.70-, 0.51-, and 0.49-fold for downregulated proteins. For those proteins that were significantly regulated at 6 h, only 7 showed a difference of 2-fold or greater and these were all upregulated under purine-deplete conditions (Fig. 1). With prolonged purine restriction, progressively more proteins demonstrated altered abundance, and by 48 h, the profiled proteome for purine-starved cells harbored some 841 proteins significantly changed in abundance, of which 151 were upregulated and 105 downregulated by 2-fold or greater (Table S1 and Fig. 1).

The accuracy of the AMT tag analyses for estimating modest, but significant, differences in the proteomes of purine-starved and purine-replete cells was exemplified by its comparison to an independent shotgun proteomic dataset collected after 24 h of purine starvation and evaluated by LC-MS/MS and spectral counting (see Text S1 and Table S2). Comparison of the 24 h AMT tag and spectral counting datasets revealed 1877 common proteins (see Table S2), and of the 301 proteins significantly upregulated or downregulated (p-value ≤0.05) at 24 h by the AMT tag method (Table S1 and Fig. 1), 202 followed a similar trend in the spectral counting dataset, where 110 proteins were downregulated and 92 were upregulated (Table S2).

#### Temporal responses within the purine pathway proteome of purine-starved leishmania

**Figure 2:**
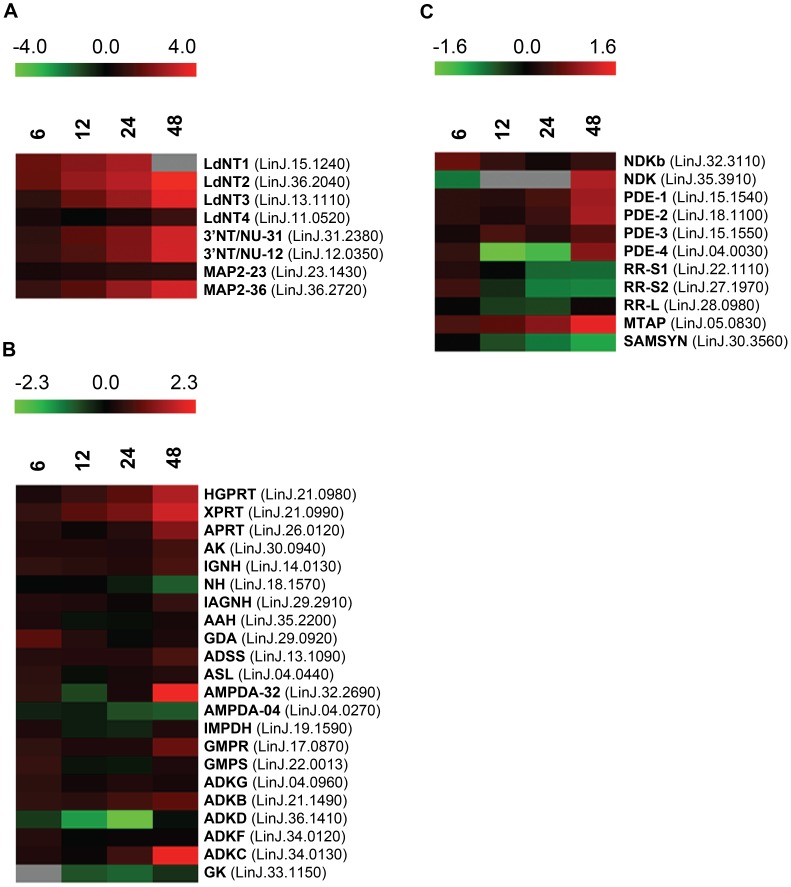
Heat maps depicting temporal changes in purine metabolism during purine starvation. Heat maps were generated using the open-source analysis software Multi Experiment Viewer MeV v4.6 (http://www.tm4.org/mev/MeV_4_6) [150] to show the comparative log_2_ abundance ratios between purine-replete and purine-starved samples at 6, 12, 24, and 48 h. The log_2_ scale for each heat map is shown on the bar above. TriTrypDB accession numbers (http://tritrypdb.org/tritrypdb/) are included on the right of each panel. Upregulated proteins are depicted by red bars, downregulated proteins by green bars, and missing data points by grey bars. (A) Log_2_ abundance ratios for cell surface purine salvage components. (B) Log_2_ abundance ratios for various intracellular purine salvage pathway components. (C) Log_2_ abundance ratios for other intracellular purine metabolizing enzymes. Abbreviations: LdNT1-4, *L. donovani* nucleobase/nucleoside transporters 1–4; 3′NT/NU-31 and 3′NT/NU-12, 3′-nucleotidase/nucleases corresponding to sequences on chromosomes 31 and 12, respectively; MAP2-23 and MAP2-36, membrane acid phosphatases corresponding to sequences on chromosomes 23 and 36, respectively; HGPRT, hypoxanthine phosphoribosyltransferase; XPRT, xanthine phosphoribosyltransferase; APRT, adenine phosphoribosyltransferase; AK, adenosine kinase; IGNH, inosine-guanosine nucleoside hydrolase; NH, nonspecific nucleoside hydrolase; IAGNH, purine-specific nucleoside hydrolase; AAH, adenine aminohydrolase; GDA, guanine deaminase; ADSS, adenylosuccinate synthetase; ASL, adenylosuccinate lyase; AMPDA-32 and AMPDA-04, putative adenosine monophosphate (AMP) deaminases corresponding to sequences on chromosomes 32 and 4, respectively; IMPDH, inosine monophosphate dehydrogenase; GMPR, guanosine monophosphate (GMP) reductase; GMPS, GMP synthase; ADKG, ADKB, ADKD, ADKF, ADKC, multiple adenylate kinase activities; GK, putative guanylate kinase; NDKb and NDK, nucleoside diphosphate kinases; PDE-2, cAMP specific phosphodiesterase A; PDE-1, -3, -4, putative cAMP phosphodiesterases; RR-S1 and RR-S2, putative ribonucleoside diphosphate reductase small chains; RR-L, putative ribonucleoside diphosphate reductase large chain; MTAP, putative methylthioadenosine phosphorylase; and SAMSYN, S-adenosylmethionine synthetase.

**Figure 3:**
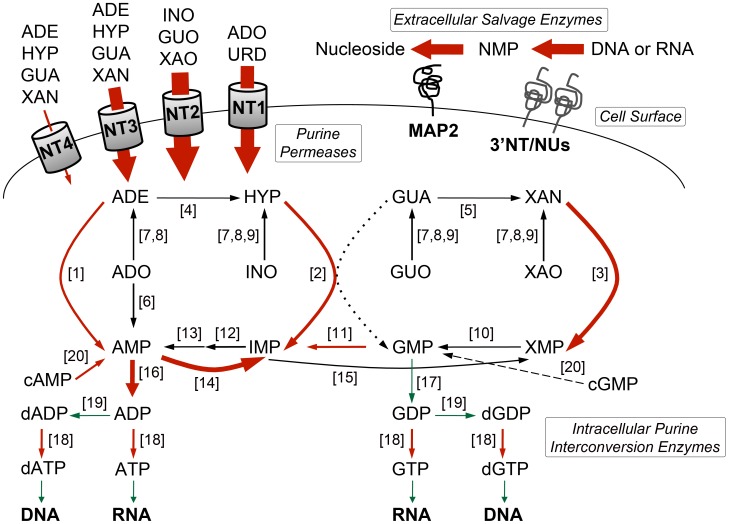
Purine acquisition and interconversion in *Leishmania* 48 h post induction of purine starvation. The thickness of the arrows represents the magnitude of upregulation in the 48-fold or more are shown in red and those that are downregulated are shown in green. Dashed arrow indicates that the conversion of cGMP to GMP is predicted but has yet to be demonstrated in *Leishmania*. Dotted arrow indicates that the conversion of GUA to GMP by HGPRT is insubstantial in *L. donovani* [25]. *Abbreviations*: NT1, LdNT1; NT2, LdNT2; NT3, LdNT3; NT4, LdNT4; ADE, adenine; HYP, hypoxanthine; GUA, guanine; XAN, xanthine; ADO, adenosine; INO, inosine; GUO, guanosine; XAO, xanthosine; URD, uridine; XMP, xanthosine monophosphate; NMP, nucleoside monophosphate; ADP, adenosine diphosphate; GDP, guanosine diphosphate; dADP, deoxyadenosine diphosphate; dGDP, deoxyguanosine diphosphate; ATP, adenosine triphosphate; GTP, guanosine triphosphate; dATP, deoxyadenosine triphosphate; dGTP, deoxyguanosine triphosphate; for all other abbreviations see the legend of Fig. 2. *Activities* (*see the legend of* *Fig. 2* *for abbreviations*): [1] APRT; [2] HGPRT; [3] XPRT; [4] AAH; [5] GDA; [6] AK; [7] NH; [8] IAGNH; [9] IGNH; [10] GMPS; [11] GMPR; [12] ADSS; [13] ASL; [14] AMPDA; [15] IMPDH; [16] various ADK; [17] GK; [18] NDKb and NDK; [19] ribonucleoside diphosphate reductase; [20] various cyclic nucleotide phosphodiesterases.

Some of the earliest and most striking changes in the proteome following purine removal involved the upregulation of purine salvage components located at the cell surface. These included the purine nucleoside transport proteins, **LdNT1** (LinJ.15.1230-50) and **LdNT2** (LinJ.36.2040), which participate in adenosine and 6-oxopurine nucleoside acquisition, respectively [51], [52], the purine nucleobase transporter **LdNT3** (LinJ.13.1110) [53], and the membrane bound 3′NT/NUs (LinJ.12.0350 and LinJ.31.2380) and acid phosphatases (**MAP2**) (LinJ.36.2720) that digest nucleic acids and mononucleotides down to their respective nucleosides [13], [54]–[58] (Figs. 2A and 3). Thus, some of the earliest changes appear tailored to enhance purine acquisition at the cell surface. By 12–24 h, the levels of two of the major intracellular purine salvage enzymes, xanthine phosphoribosyltransferase (**XPRT**) (LinJ.21.0990) and hypoxanthine-guanine phosphoribosyltransferase (**HGPRT**) (LinJ.21.0980) [25], were also significantly (but modestly) increased. These enzymes catalyze the conversion of xanthine and hypoxanthine to XMP and IMP, respectively, and provide a major route of conversion for all purine nucleobases to the nucleotide level in these parasites (Figs. 2B and 3 and refs. [21], [25], [59], [60]). By 48 h, more changes were evident in the pathway, with increases observed for the purine nucleobase transporter **LdNT4** (LinJ.11.0520) [61], and the purine salvage and interconversion enzymes, adenine phosphoribosyltransferase (**APRT**) (LinJ.26.0120), guanosine monophosphate reductase (**GMPR**) (LinJ.17.0870), adenylosuccinate synthetase (**ADSS**) (LinJ.13.1090), one of the two profiled adenosine monophosphate deaminases (**AMPDA-32**) (LinJ.32.2690), adenosine kinase (**AK**) (LinJ.30.0940), purine-specific nucleoside hydrolase (**IAGNH**) (LinJ.29.2910), and the 6-hydroxypurine nucleoside hydrolase (**IGNH**) (LinJ.14.0130) (Figs. 2B and 3). By contrast, no significant change, and in one case even decreased abundance, was observed for guanine deaminase (**GDA**) (LinJ.29.0920), the nonspecific nucleoside hydrolase (**NH**) (LinJ.18.1570), adenylosuccinate lyase (**ASL**) (LinJ.04.0440), GMP synthase (**GMPS**) (LinJ.22.0013), adenine aminohydrolase (**AAH**) (LinJ.35.2200), the alternative AMPDA (**AMPDA-04**) (LinJ.04.0270), and IMP dehydrogenase (**IMPDH**) (LinJ.19.1590). Although these later abundance changes within the pathway at 48 h were modest (the majority being ∼2-fold or less) the cumulative effect of these changes would seem to suggest that flux through the pathway is retooled to favor adenylate nucleotide production (Fig. 3). This supposition is supported by the following: that two putative adenylate kinase orthologs (**ADKB** and **ADKC**) (LinJ.21.1490 and LinJ.36.1410), one of which (ADKB) has a flagellar location in *Trypanosoma brucei* [62], were significantly upregulated in purine-starved cells, whereas the reciprocal activity within the guanylate branch of the pathway, guanylate kinase (**GK**) (LinJ.33.1150), was decreased at the protein level (Figs. 2B and 3); that a putative methylthioadenosine phosphorylase (**MTAP**) (LinJ.05.0830) that catalyzes the conversion of methylthioadenosine (**MTA**) to adenine and 5-deoxy-5-(methylthio)ribose-1-phosphate [63] was upregulated, whereas the production of S-adenosylmethionine (**SAM**) from ATP and methionine by SAM synthetase (**SAMSYN**) (LinJ.30.3560) [64], [65] was disfavored (Fig. 2C); and that a spectrum of phosphodiesterases (**PDE**) (LinJ.15.1540-50, LinJ.18.1100, and LinJ.04.0030) that liberate AMP, and possibly GMP, from their respective cyclic nucleotides [66]–[68] were also upregulated at the protein level (Figs. 2C and 3). Finally, in addition to the other changes in the purine pathway, the formation of deoxyribonucleotides for DNA synthesis, unsurprisingly, also appeared suppressed in growth-arrested, purine-restricted cells, since the amounts of both the large and small subunits of ribonucleotide reductase (LinJ.22.1110, LinJ.27.1970, LinJ.28.0980) [69], [70] were decreased (Figs. 2C and 3).

#### Global proteome remodeling in purine-starved cells

**Figure 4:**
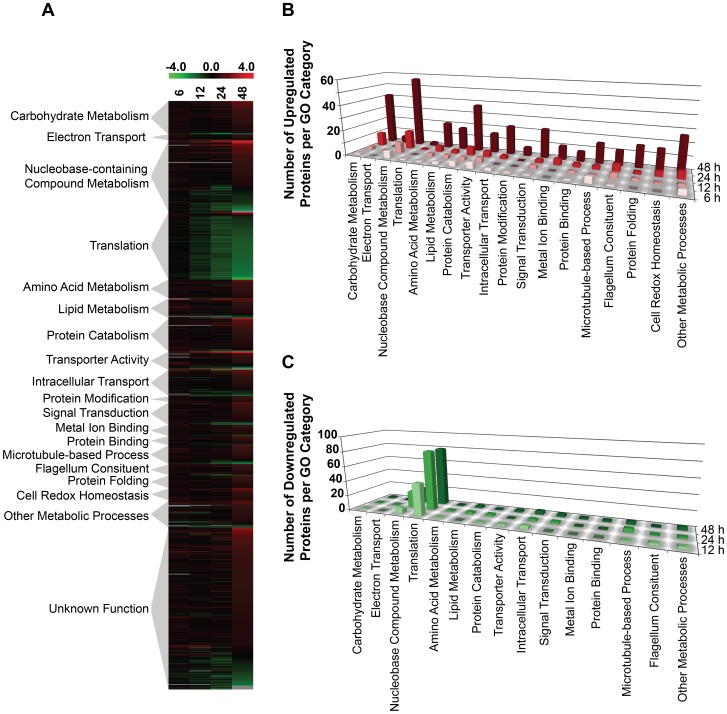
Global proteome remodeling in purine-starved cells. 777 proteins that were significantly changed (p-value ≤0.05) and either up- or downregulated by a log_2_ value of 0.5 or more at 6, 12, 24, and 48 h were grouped according to their molecular function. (A) Heat map illustrating the temporal changes between purine-replete and purine-starved samples at 6, 12, 24, and 48 h. The log_2_ scale is shown on the bar above. Missing data points for particular proteins within the time-course are depicted by the grey bars. Proteins were sorted according to molecular function and the heat map generated using the open-source analysis software Multi Experiment Viewer MeV v4.6 (http://www.tm4.org/mev/MeV_4_6) [150]. (B) and (C) 3-dimensional bar graphs showing the number of either upregulated (B) or downregulated (C) proteins in each functional category. Both the heat map and bar graphs were generated using the data in Table S3.

We next asked whether other global changes in the proteome could be linked to the observed morphological and metabolic changes observed upon purine withdrawal from the media [8]. Proteins that were significantly altered (p-value of ≤0.05) by a log_2_ abundance ratio of either ≥0.5 or ≤−0.5 (which corresponds to either a 1.4-fold upregulation or more, and a 0.7-fold downregulation or less) in purine-starved parasites were classified in terms of their biological and metabolic functions (see Fig. 4 and Table S3). A total of 777 proteins were analyzed and 561 were categorized according to their GO category and molecular function, but the remaining 216 were of unknown function and therefore, could not be classified. The large number of unclassified proteins (∼28%) is reflective of the prevalence of hypothetical proteins within the annotated *L. infantum* genome [49].

**Figure 5:**
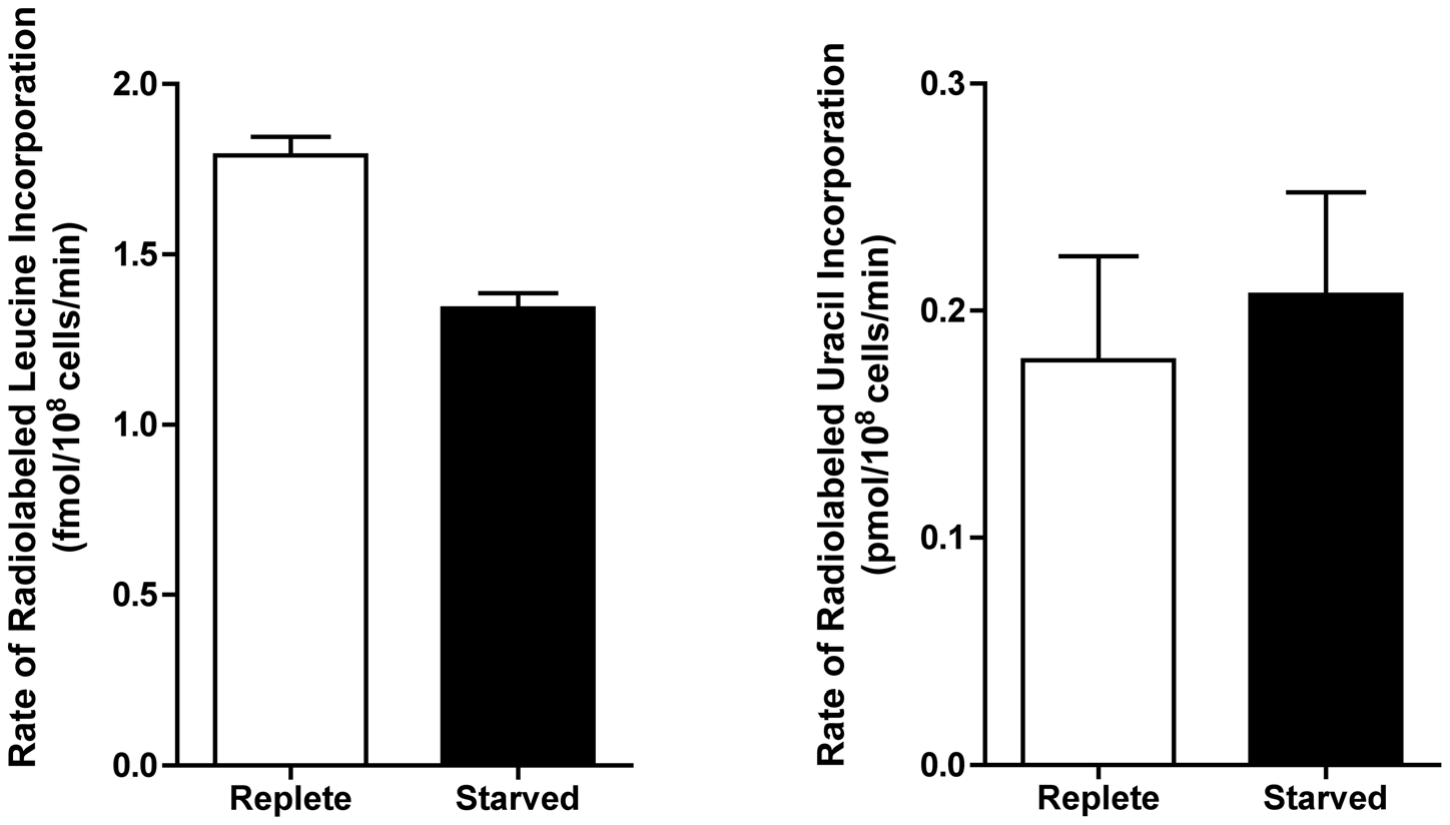
Rate of incorporation of radiolabeled leucine and uracil into purine-starved and purine-replete cells. The rate of incorporation of [4,5-^3^H]-leucine and [2-^14^C]-uracil was compared between purine-replete (open bars) and cells starved for purine for 24 h (closed bars). Rates were calculated based upon 3 biological replicates per time-point for each condition (purine-replete *versus* purine-starved) and the data represent the mean rate of incorporation from two independent assays.

The earliest changes (6 h) in the proteome predominantly involved the upregulation of transporter proteins, as well as catabolic activities involved in protein and nucleic acid digestion, with the latter category largely populated with digestive activities involved in purine acquisition at the cell surface (Figs. 2A and 4B and Table S3). By 12–24 h, however, a diverse array of proteins was significantly upregulated, including proteins involved in intracellular purine interconversion (namely HGPRT and XPRT), carbohydrate metabolism (specifically involved in gluconeogenesis and NADPH production in the oxidative branch of the pentose phosphate pathway), signal transduction, flagella motility and structure, protein folding, and cellular redox response and homeostasis (Fig. 4B and Table S3). Proteome restructuring was also facilitated at 12–24 h by the downregulation of certain cellular constituents (Fig. 4C and Table S3). Predominant amongst the downregulated proteins were ribosome components, as well as other factors involved in protein synthesis, signifying that translation is reduced in purine-starved cells as early as 12 h post purine removal from the medium. Analysis of [^3^H]-leucine incorporation into the trichloroacetic acid-precipitable pool of cells starved for purine for 24 h indicated that the rate of incorporation was reduced by ∼25% in comparison to cells grown continuously in hypoxanthine (Fig. 5), signifying that protein synthesis is downregulated during purine starvation. In contrast, the rate of incorporation of [2-^14^C]-uracil into the same trichloroacetic acid-precipitable pool was effectively equivalent between purine-starved and purine-replete cells over the same time course (Fig. 5). Proteins involved in nucleic acid metabolism were also significantly reduced in purine-starved cells. Specifically those involved in nucleic acid replication, processing, and repair, including ribonucleotide reductase, RNA helicases, and DNA topoisomerases, as well as various DNA and RNA binding proteins, including two universal minicircle sequence binding protein (**UMSBP**) orthologs (LinJ.36.1680 and LinJ.36.1720), which have been implicated in kinetoplast DNA (**kDNA**) replication and mitochondrial and nuclear segregation in trypanosomes [71]. Remarkably, very few other metabolic activities were downregulated in purine-starved cells at 12–24 h, but amongst the downregulated activities were proline dehydrogenase (alternatively named proline oxidase) (LinJ.26.1590) and UDP-sugar pyrophosporylase (LinJ.17.1260), which participate in proline catabolism [72] and glycan precursor biosynthesis [73], respectively.

By 48 h the proteome of purine-starved cells was extensively changed. Although many of the changes that were detected were subtle, involving changes of two-fold or less (Fig. 1), many of the altered proteins could be grouped to either the same metabolic pathway or were similar in their molecular function (Fig. 4 and Table S3), denoting, perhaps, a significant role for these biological processes in the adaptive response to purine stress. Proteins involved in degradative or catabolic processes were generally upregulated within the proteome of growth-arrested, purine-starved cells, including those proteins involved in fatty acid ß-oxidation, the catabolism and interconversion of amino acids, and protein and nucleic acid degradation (Fig. 4B and Table S3). In contrast, factors involved in protein and DNA synthesis, both of which are ATP-consuming reactions, remained substantially downregulated (Fig. 4C and Table S3).

Amongst the other proteome constituents upregulated, proteins involved in cell redox homeostasis and oxidant defense, intracellular trafficking, and amino acid interconversion and degradation were prevalent. However, by far the predominant functional category of upregulated proteins at 48 h was that of carbohydrate metabolism, with 42 candidates out of 360 upregulated proteins with an assigned function allocated to this category (Fig. 4 and Table S3). Specifically, those proteins associated with glycolysis and gluconeogenesis, the pentose phosphate pathway (Fig. S1), and the tricarboxylic acid (**TCA**) cycle, were all augmented. Given that many of the components of these pathways are sequestered in glycosomes in *Leishmania*, peroxisome-like organelles [74]–[76], we compared the protein abundance data at 6–48 h for all known and *in silico* predicted glycosomal proteins [77] to determine whether a reduced turnover of this organelle could account for the observed protein upregulation (Table S4). However, the data revealed little consensus at each time point, suggesting that changes in glycosome turnover likely do not contribute towards proteome remodeling during purine starvation.

**Figure 6:**
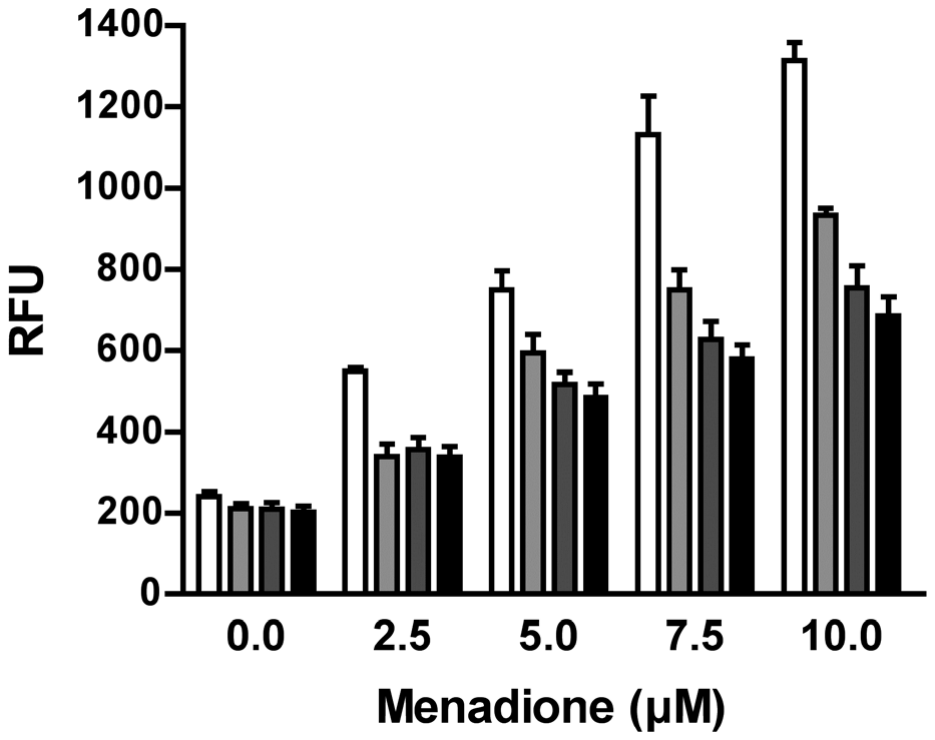
Response of purine-starved and purine-replete parasites to ROS induction. Purine-replete and purine starved promastigotes were exposed to increasing concentrations (2.5–10 µM) of the ROS-generating compound menadione. Generation of ROS was measured by incubating parasites with the cell-permeant fluorescein derivative H_2_DCFDA. The RFU attributable to ROS in 10^6^ cells are depicted for parasites grown continuously in 100 µM hypoxanthine (open bars) or starved for purine for 24 h (light grey bars), 48 h (dark grey bars), or 72 h (black bars). The data represent three independent biological replicates. (Error bars indicate standard deviation).

Since proteome profiling of purine-starved *versus* purine-replete cells revealed that multiple factors involved in oxidant defense were upregulated at the 24 and 48 h time points (Table S3), we also investigated whether purine-starved cells were under increased duress from reactive oxygen species (**ROS**) by employing the cell-permeant ROS indicator 2′,7′-dichlorodihydrofluorescein diacetate (**H_2_DCFDA**). While cells starved for purine for 24, 48, and 72 h did not appear to harbor increased ROS (Fig. 6), incubation of these starved cells in the presence of increasing concentrations of menadione (2.5–10 µM) revealed an enhanced capacity to deal with increased levels of ROS, and consequently, oxidative stress, which was considerably greater than that observed in cells continuously grown in hypoxanthine (Fig. 6). Moreover, the antioxidant capacity of cells purine-starved for 24, 48, and 72 h was progressively amplified. A similar phenomenon was also observed with cells starved for purine for 24 and 48 h and incubated in the presence of either 2 or 4 mM H_2_O_2_ (Fig. S2).

**Figure 7:**
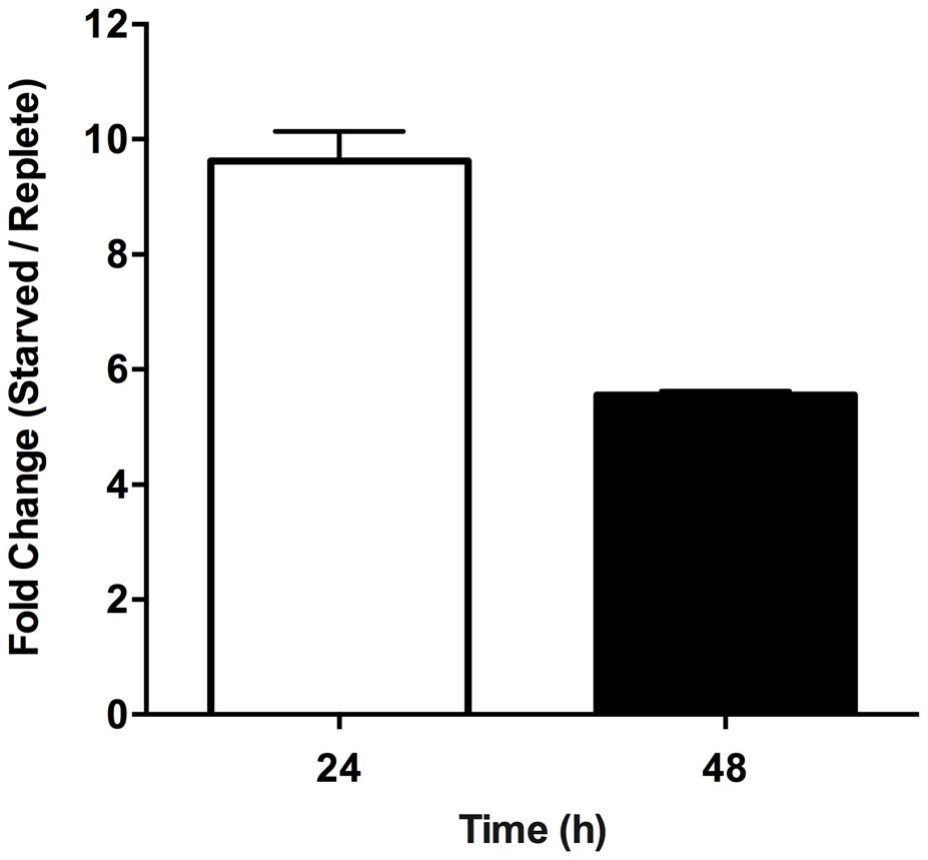
Effect of purine starvation on intracellular proline levels. The free intracellular L-proline concentration was determined for purine-replete and purine-starved cells. Bars indicate the fold change between purine-starved *versus* purine-replete parasites at 24 h (open bar) and 48 h (black bar) post purine removal. Error bars indicate standard deviation; data represent two independent biological replicates.

Within those proteins classified as participating in the catabolism and interconversion of amino acids (Table S3), one pathway appeared particularly represented, namely that of proline biosynthesis and interconversion (Fig. S3). Proteins encoding for a putative glutamate 5-kinase (LinJ.26.2740), pyrroline-5-carboxylate synthetase-like protein (LinJ.32.3340), and pyrroline-5-carboxylate reductase (LinJ.13.1420), that all participate in proline biosynthesis were modestly upregulated at 48 h, whilst proline dehydrogenase (LinJ.26.1590), which is involved in proline catabolism, was significantly reduced at 12, 24 and 48 h post purine removal (Table S3, and Fig. S3). Proline is a key stress response metabolite in a number of organisms and, although its precise role remains rather enigmatic, it has been shown to enhance cell survival during environmental stress [78]–[82]. Significantly, in *T. cruzi*, a parasite highly related to *Leishmania*, proline has emerged as an important nutrient in combating environmental stress [79] and is vital during metacyclogenesis, a process that has also been linked with nutrient stress [18], [83]. Thus, we investigated whether proline levels were also augmented in *L. donovani* during purine starvation. A marked increase in intracellular proline was observed in purine-starved cells at 24 h and 48 h (9.7- and 5.7-fold, respectively) in comparison to those cells grown in hypoxanthine (Fig. 7). These data confirm the veracity of the proteomics results, and significantly, suggest that small, but cumulative changes at the protein level for multiple enzymes within the same pathway can lead to a significant modulation at the metabolite level.

Proteome remodeling, particularly during differentiation of the procyclic or insect stage of *Leishmania* to the mammalian infectious amastigote form, has also been described through autophagy [16], [84]–[86]. Whether autophagy is also involved in proteome remodeling during purine deprivation is unclear. While purine-starved cells by 48 h had substantially boosted protein digestive activities, including cysteine peptidase A (LinJ.19.1460), which has also been implicated in autophagosome degradation [16], most of the described canonical autophagic machinery [16], [87], [88] was either not upregulated or could not be profiled within the proteomics dataset (Table S1). However, vacuolar protein sorting-associated protein 4 (**VPS4**, LinJ.29.2610) [85] was upregulated 2-fold by 48 h post purine removal from the medium, an activity previously shown to be important for cytosolic autophagosome processing in *Leishmania* [85]. Perhaps more significantly, VPS4 in *Leishmania* has also been shown to be important for survival during nutrient depletion, as well as for the differentiation of promastigotes during metacyclogenesis [85]. *De novo* phospholipid biosynthesis has also been critically implicated in autophagosome formation during autophagy [89], since phospholipids are core components of the autophagosome membrane bilayer, and phosphatidylethanolamine (**PE**), in particular, also functions to tether Autophagy-related protein (**Atg8**) to the autophagosome membrane, a crucial step in autophagosome formation and expansion [88]–[90]. At 48 h in purine-starved cells the proteomics data suggested that PE formation is favored, since both ethanolamine-phosphate cytidylyltransferase (LinJ.32.0940) and sphingosine 1-phosphate lyase (LinJ.30.2360) are augmented (Fig. S4 and Table S3). Note that the degradation of sphingolipids by sphingosine 1-phosphate lyase provides the major route for phosphoethanolamine synthesis in *Leishmania* [91]. In addition, the synthesis of the core phospholipid phosphatidylcholine from PE also appeared enhanced, since phosphatidylethanolamine-methyltransferase-like protein levels (LinJ.31.3250 and LinJ.31.2360) were also elevated (Fig. S4 and Table S3).

Reconfiguration of the proteome during purine stress requires the removal of existing molecules from various cellular compartments, as well as the redistribution of new molecules. From the proteomics data, it appeared that the capacity for intracellular transport was greatly enhanced by 48 h post purine removal from the medium (Fig. 4 and Table S3). Amongst the upregulated proteins at 48 h were a number of factors involved in intracellular vesicle formation, fusion, and exocytosis, as well as an array of kinesins and dyneins that likely facilitate the movement of these vesicles along the microtubular network. Notably, two polypeptides encoding kinesin K39 (LinJ.14.1600 and LinJ.14.1190) and a putative kinesin (LinJ.16.1550) were not amongst these upregulated molecular motors at 48 h, although LinJ.14.1190 and LinJ.16.1550 were initially upregulated at 6 h. By contrast, these kinesins were significantly downregulated at 24–48 h and, given that kinesin K39 (LinJ.14.1190), at least, is apparently enriched at the posterior poles of the cortical cytoskeleton in a cell cycle-dependent manner during cytokinesis [92], their downregulation would seem to correlate with the arrest in growth of the purine-starved cells.

### Analysis of the Molecular Mechanisms Underlying Proteome Restructuring

#### Whole transcriptome analysis of purine-starved and purine-replete cells

**Figure 8:**
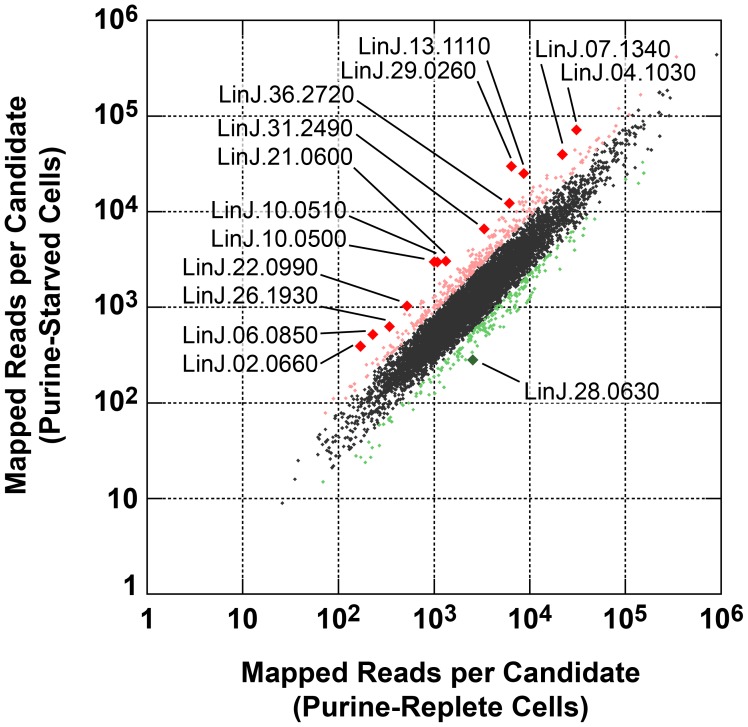
Scatter plot of SL RNA-Seq data comparing mRNA abundance between purine-replete and purine-starved cells. The number of reads mapped to individual mRNA sequences (see Table S5) were compared between cells grown in medium supplemented with 100 µM hypoxanthine to those cultivated in medium without purine supplementation for 24 h. Those mRNAs changed by less than 2-fold are denoted by small black diamonds; mRNAs upregulated 2- to 4-fold are denoted by small pink diamonds; mRNAs downregulated 2- to 4-fold are denoted by small green diamonds; mRNAs upregulated by 4-fold or more are denoted by large red diamonds; mRNAs downregulated by 4-fold or more are denoted by large green diamonds. The TriTrypDB [50] accession numbers for those mRNAs most significantly changed are shown.

**Figure 9:**
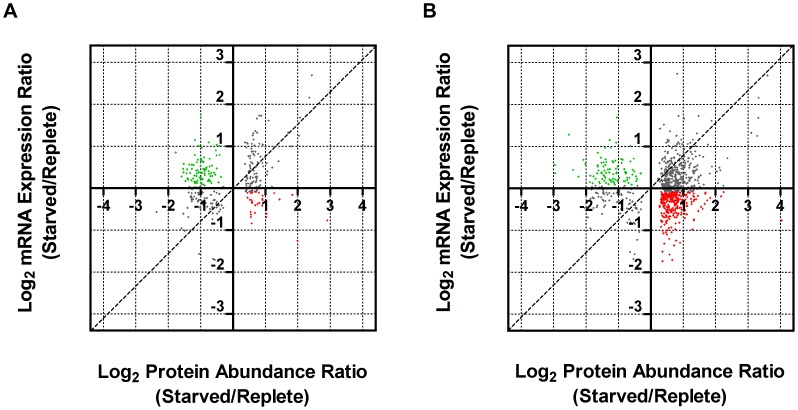
Comparison of fold changes at the protein and mRNA level in purine-starved cells. Proteins were sorted by log_2_ abundance ratio at both 24 h (A) and 48 h (B) and plotted against the log_2_ expression ratio at 24 h for the corresponding mRNA as measured by SL RNA-seq. Dashed lines indicate an exact correlation between the changes at the protein and mRNA level. Grey dots indicate those proteins that exhibit a similar trend at the mRNA level (upregulated, upper right quadrant, and downregulated, lower left quadrant), green dots correspond to those proteins that were downregulated but where the corresponding mRNA was upregulated (upper left quadrant), and red dots correspond to those proteins that were upregulated but where the corresponding mRNA was downregulated (lower right quadrant).

*Leishmania* exhibit unusual mechanisms of gene regulation. The majority of the genome is constitutively transcribed by RNA polymerase II as long polycistrons (pre-mRNAs that contain multiple coding sequences) that are *trans*-spliced into mature mRNAs by the coordinated addition of a 5′ capped 39 ribonucleotide splice leader (**SL**) to the 5′ untranslated region (**UTR**) and a polyadenylate tail to the 3′ UTR of each mRNA [36], [93]. Thus, in these parasites, classical transcriptional regulation is absent; instead, changes in protein abundance can arise from changes at the mRNA level, involving alterations in mRNA abundance, *trans*-splicing, and polyadenylation, or from changes at the translational or post-translational level. As a first step towards understanding the molecular mechanisms underlying proteome modification upon purine restriction, Whole Transcriptome Shotgun Sequencing (**RNA-seq**) was undertaken to quantitate mRNA abundance differences between purine-starved and purine-replete cells. Since the RNA-seq libraries were constructed using an SL-specific primer for 2^nd^ strand cDNA synthesis (see *Materials and Methods*), this also enabled us to determine whether alternative *trans*-splice sites were used under conditions of purine restriction [39], [40], [42]–[45], [94]. A total of 53,249,393 and 27,430,972 reads were gathered from the purine-replete and purine-starved samples, respectively. (The SL RNA-Seq data from this study have been submitted to the NCBI Gene Expression Omnibus (**GEO**) database at http://www.ncbi.nlm.nih.gov/geo/ under the accession no. GSE48394). These reads were mapped to 8185 genes of which 8075 had at least 100 reads across both conditions and accounted for ∼96% coverage within the reference *L. infantum* genome [49] (Table S5 and Fig. 8). The median number of reads from purine-replete cells was 2,522 and from purine-starved cells was 1,132. After normalization of the data (see *Materials and Methods*), the number of mRNAs that were changed in abundance in purine-starved cells by two-fold or more (log_2_ expression ratio ≥1 or ≤−1) at 24 h was 523 (Table S5 and Fig. 8). Of these regulated mRNAs, 324 were upregulated and 199 were downregulated. However, in general no substantial changes in the primary *trans*-splice sites used for each mRNA were observed between purine-replete and purine-starved cells (the splice site data is available at http://www.ncbi.nlm.nih.gov/geo/ under the accession no. GSE48394), indicating that where differences in mRNA abundance were observed they were unlikely due to differential mRNA processing in these organisms. Of the regulated mRNAs, 13 were upregulated by 4-fold or more, and significantly amongst these were the mRNAs for two cell surface purine activities *LdNT3* and *MAP2-36*, which were upregulated by 6.5 and 4.5-fold, respectively. A comparison of the mRNA levels for the key purine pathway components that were regulated at the protein level at 24 and 48 h post purine restriction (Fig. S5 and Table 1) indicated that the fold-changes at the mRNA and protein level for the most part tracked together, although there were two notable exceptions (LdNT1 and LdNT2) where changes at the mRNA level correlated poorly with the fold changes at the protein level. These nucleoside transporters were highly (6- to 16-fold) upregulated at the protein level at 24 and 48 h, but showed only a modest change at the mRNA level (*LdNT1*) or an apparent decrease (*LdNT2*) at 24 h. Comparison of the SL RNA-seq data to the entire proteome datasets at 24 and 48 h showed considerable discordance between the changes manifested at the protein and mRNA level in purine-starved cells (Fig. 9 and Table S6). Specifically, there were a number of downregulated proteins where the corresponding mRNA was elevated in abundance (Fig. 9 A and B, upper left quadrant and Table S6) and upregulated proteins where the corresponding mRNA was downregulated (Fig. 9 A and B, lower right quadrant). These correlations were consistent between the 229 mRNA and protein pairs present in the 24 and 48 h proteome data, where only one protein and mRNA pair (a putative ethanolamine-phosphate cytidylyltransferase, LinJ.32.0940) showed a decreased mRNA and protein abundance at 24 h but increased protein abundance at 48 h (see Table S6 for the matched protein and mRNA data). These data suggest that, in addition to changes in mRNA abundance, proteome remodeling in response to purine stress is likely orchestrated by translational and post-translational mechanisms.

**Table 1:** Comparison of relative mRNA abundance change during purine starvation by SL RNA-seq and qRT-PCR.

Descriptor, Accession Number	mRNA Fold	Change (24 h)	Protein	Fold Change
	SL RNA-seq	qRT-PCR	24 h	48 h
**LdNT1.1**, LinJ.15.1230-50	1.79	2.27±0.40	6.48	7.66^b^
**LdNT2**, LinJ.36.2040	0.59	1.07±0.41	7.46	16.45
**LdNT3**, LinJ.13.1110	6.45	5.75±0.23	5.39	12.13
**LdNT4**, LinJ.11.0520	0.97	1.70±0.44	1.34	2.03
**3′NT/NU-12**, LinJ.12.0350	3.20	5.07±0.61	4.19	9.97
**3′NT/NU-31**, LinJ.31.2380	1.48	5.06±0.05	4.11	9.71
**MAP2-36**, LinJ.36.2720	4.47	4.81±0.45	5.12	10
**XPRT**, LinJ.21.0990	3.05	1.97±0.19	2.13	3.68
**AAT19**, LinJ.07.1340	3.71	3.52±0.47	1.04	1.15
**La RNA binding protein**, LinJ.21.0600	5.10	1.13±0.05	0.75	0.85
**Oxidoreductase**, LinJ.29.0260	10.34	4.29±0.78	ND*	ND*
**Hypothetical**, LinJ.31.2490	4.41	3.67±0.31	ND*	1.27
**Hypothetical**, LinJ.04.1030	5.21	2.15±0.69	ND	ND
**Hypothetical**, LinJ.28.0630	0.25	1.04±0.06	ND	ND
**SHERP**, LinJ.23.1210, LinJ.23.1230	1.08±0.02	1.62±0.11	ND	ND
**UMPS** a, LinJ.16.0560	0.91	1.00^a^	1.05	1.12

qRT-PCR data represents the mean fold change ± standard deviation from two independent biological replicates. ND, not detected in replete or starved sample set; ND* not detected in replete samples only;

a serves as an internal control normalized to 1.00 for each qRT-PCR assay;

b since LdNT1.1 peptides were not detected in the 48 h replete sample, the fold change was calculated by comparing the combined average AMT tag intensity recorded at 6, 12, and 24 h (replete samples, n = 11) with the average AMT tag intensity at 48 h (starved samples, n = 5).

#### Establishing whether translational mechanisms lead to the augmentation of select candidates within the purine-starved proteome

RNA-seq is particularly suited for the quantitative analysis of transcript expression levels due to the massive amounts of sequence data and the number of reads that can be generated for each mRNA [38], [39], [41]–[45], especially in a genome as small as that of *L. donovani* [49], [95]. Nevertheless, it was still important to confirm the fold-changes observed at the mRNA level in purine-starved cells for some of the most regulated mRNAs and proteins identified in these analyses. Thus, the levels for those mRNAs described in Table 1 were quantified from purine-replete and purine-starved cells at 24 h by quantitative reverse transcriptase PCR (**qRT-PCR**). In general, the mRNA abundance changes measured by SL RNA-seq or qRT-PCR at 24 h (Table 1) were in good agreement, confirming the utility of RNA-seq for profiling mRNA abundance changes during purine starvation. The results were also comparable with our previous qRT-PCR analyses that established mRNA levels at 48 h for the purine permeases and key purine pathway components between purine-starved and purine-replete cells [8]. There were, however, a few notable discrepancies between the qRT-PCR and SL RNA-seq data. In particular, the mRNA level for the La RNA binding protein (LinJ.21.0600) was significantly increased in purine-starved cells when measured by SL RNA-seq, but was not appreciably elevated when measured by qRT-PCR. While the cause of this discrepancy likely reflects the inherent differences between the two methodologies, it is noteworthy that the abundance of the La RNA binding protein was not augmented, but rather was decreased in purine-starved cells at the proteome level.

In most cases, the abundance changes for those subset of proteins listed in Table 1, as determined by proteomic analysis, corresponded closely to the changes in mRNA abundance determined by the SL RNA-seq and qRT-PCR analyses, implying that the regulation of these proteins during purine stress was predominantly mediated at the level of mRNA abundance. In contrast, the abundance of LdNT1.1 and LdNT2 proteins was significantly more augmented than the changes wrought at the mRNA level, where the increase was modest for *LdNT1.1* mRNA and non-existent for *LdNT2* mRNA, intimating that regulation occurs via translational and/or post-translational mechanisms during purine stress. Similarly, the incremental increase observed for LdNT3 protein in our proteomics analysis between 24–48 h time points was not reflected by our combined qRT-PCR analysis of *LdNT3* mRNA abundance at these same time points (Table 1; ref. [8]), suggestive of an additional level of regulation at either the translational or post-translational level.

To investigate the contribution of translational mechanisms to proteome remodeling during purine stress, we used a novel Dual-Luciferase reporter system in which the firefly luciferase gene (***Fluc***) (KF035118) was integrated in place of the coding sequence of one allelic copy of the gene of interest in a manner such that the native 5′ and 3′ UTRs remained intact. This approach conserves the sites of *trans*-splicing and polyadenylation, as well as any potential *cis*-acting elements in the UTRs of each mRNA, which is of particular importance in *Leishmania* and other kinetoplastid parasites where it has been demonstrated that regulation of mRNA abundance and translation is often mediated by *cis*-acting elements encoded in the 5′ and/or 3′ UTRs [96]–[101]. A *Renilla* luciferase gene (***Rluc***) (KF035116) integrated in place of one copy of *L. donovani* UMP synthase (***UMPS***) (LinJ.16.0560) [102], also referred to as orotidine-5-phosphate decarboxylase/orotate phosphoribosyltransferase, was used as a control to normalize the luciferase activity between experiments. Note that from our current and previous qRT-PCR analyses, western analyses, as well as from the SL RNA-seq and proteomics data described here, *UMPS* mRNA and protein levels do not appear to change significantly in response to purine stress (Tables 1, S1, and S5, and ref. [8]).

For each cell line with an integrated *Fluc* construct, both Fluc activity and *Fluc* mRNA levels were assessed to distinguish the contributions of mRNA abundance and translational mechanisms to reporter regulation in response to purine stress. The mRNA and protein abundance of the purine nucleobase transporter LdNT4 was not substantially changed following 24 h purine starvation (Table 1), and this was directly mirrored by the activity and mRNA abundance of the *Fluc* reporter integrated at the *LdNT4* locus (Table 2), indicating that *Fluc* by itself does not affect mRNA abundance or confer a response to purine stress. In contrast to LdNT4, the change in mRNA abundance for the *Fluc* reporter arising from its integration into the locus of corresponding purine-responsive genes listed in Table 2 was two- to four-fold lower than the changes observed for the native mRNA, suggesting that the coding sequence (**CDS**) of these purine-responsive genes contributes to the regulation of mRNA abundance. The change in Fluc activity for those reporters integrated in place of one gene copy of *3′ NT/NU-12*, *3′ NT/NU-31*, and *MAP2-36*, which encode some of the most upregulated proteins detected within the proteome of purine-starved cells (Fig. 2A), was commensurate with the change (∼1.5 to 2-fold) at the corresponding *Fluc* mRNA level (Table 2). This concordance was also observed for the native locus, where the magnitude of change for the corresponding mRNA and protein was essentially equivalent. These data support a model whereby the regulation of these genes is primarily manifested at the mRNA level, and is bestowed, at least in part, through sequences in the 5′ or 3′ UTR. For the purine permeases (LdNT1.1, LdNT2, and LdNT3) and oxidoreductase (LinJ.29.0260), despite only modest changes, and in the case of LdNT2 even decreased abundance for the *Fluc* reporter mRNA, the increase in Fluc activity was significantly higher (∼2.5 to 12-fold), indicating that native 5′ and 3′ UTRs are involved in the augmentation of their translation in response to purine stress. While translational and/or post-translational regulation was predicted for the purine permeases based on the discordance between mRNA and protein abundance data (see Table 1), the absence of protein abundance data for the oxidoreductase made such a prediction impossible. This highlights the utility of the Dual-Luciferase system for distinguishing regulatory mechanisms for candidates in which protein abundance data is either lacking or scant. Overall, the combination of proteomics, SL RNA-seq, and Dual-Luciferase reporter data revealed that regulation of gene expression in response to purine stress for several of the most upregulated proteins occurs at the level of mRNA abundance (3′NT/NU-12, 3′NT/NU-31, and MAP2-36), translation (LdNT2), or both (LdNT1.1, LdNT3, and oxidoreductase). It is also likely that post-translational mechanisms are also involved in purine stress-induced proteome remodeling, but this prospect has yet to be directly investigated.

**Table 2:** An assessment of the role of 5′ and 3′ UTRs from select purine-responsive candidates on mRNA abundance and translational regulation.

Descriptor, Accession Number	Fold Change (24 h)		
	Fluc Activity	qRT-PCR *Fluc*	qRT-PCR Gene
**LdNT1.1**, LinJ.15.1230-50	4.04±0.26	0.97±0.20	3.05±0.11
**LdNT2**, LinJ.36.2040	3.01±0.04	0.25±0.02	1.16±0.20
**LdNT3**, LinJ.13.1110	10.54±1.13	2.52±0.98	7.40±1.48
**LdNT4**, LinJ.11.0520	1.25±0.19	1.01±0.04	1.11±0.13
**3′NT/NU-12**, LinJ.12.0350	2.50±0.06	1.97±0.03	5.98±0.37
**3′NT/NU-31**, LinJ.31.2380	1.93±0.04	1.47±0.29	3.16±0.52
**MAP2-36**, LinJ.36.2720	2.21±0.04	1.85±0.15	3.73±0.46
**Oxidoreductase**, LinJ.29.0260	5.78±0.46	2.07±0.38	4.60±0.65

*L. donovani* cell lines were generated in which a *Fluc* reporter was integrated in place of one allele of the indicated locus; each *Fluc* reporter line also contained an *Rluc* reporter integrated at the *UMPS* locus as an internal normalization control. Changes in Fluc activity and mRNA abundance (qRT-PCR *Fluc*), and mRNA abundance of the corresponding endogenous allele (qRT-PCR Gene) following 24 h purine starvation were determined in parallel from aliquots of the same culture. All qRT-PCR data were normalized to *UMPS*. The mean and standard deviation determined from two independent biological replicates is shown for each analysis.

Since the purine permeases LdNT1-3, the cell surface 3′NT/NUs, and MAP2-36 are some of the earliest regulated proteins detected within the global proteome (Fig. 2A), the activity arising from the *Fluc* reporter integrated at each of these loci was compared between purine-starved versus purine-replete cells at 6 h post purine removal from the medium (Table 3). For each of these candidates, the mechanisms of regulation at 6 h and 24 h were identical (Tables 2 and 3), demonstrating that regulatory mechanisms affecting mRNA abundance and translation are invoked early in the response to purine stress. Interestingly, the mRNA level of the *Fluc* reporter integrated at the *LdNT3* locus was reduced over 2-fold at 6 h but increases 2.5-fold by 24 h of purine stress, implying the early destabilization of the *Fluc* mRNA is later reversed by a mechanism that stabilizes the mRNA. This biphasic temporal response was not observed for the wild type *LdNT3* allele, suggesting a role for the *LdNT3* CDS in protecting *LdNT3* mRNA from the general decrease in total RNA abundance that accompanies purine starvation (8). In contrast, the expression of the oxidoreductase, which was increased at both the mRNA and translational levels after 24 h purine stress, was essentially unchanged after 6 h.

**Table 3:** Elucidation of the molecular mechanisms in the early response to purine starvation.

Descriptor, Accession Number	Fold Change (6 h)		
	Fluc Activity	qRT-PCR *Fluc*	qRT-PCR Gene
**LdNT1.1**, LinJ.15.1230-50	1.94±0.08	1.06±0.001	1.52±0.17
**LdNT2**, LinJ.36.2040	1.69±0.06	0.40±0.06	0.93±0.01
**LdNT3**, LinJ.13.1110	2.63±0.28	0.43±0.001	2.90±0.18
**3′NT/NU-12**, LinJ.12.0350	1.25±0.28	1.53±0.11	3.06±0.22
**3′NT/NU-31**, LinJ.31.2380	1.36±0.03	1.54±0.14	3.22±0.61
**MAP2-36**, LinJ.36.2720	1.33±0.30	0.97±0.13	1.63±0.02
**Oxidoreductase**, LinJ.29.0260	1.36±0.23	1.02±0.03	1.24±0.02

*L. donovani* cell lines were generated as described in the *Materials and Methods* and Table 2. Changes in Fluc activity and mRNA abundance (qRT-PCR *Fluc*), and mRNA abundance of the corresponding endogenous allele (qRT-PCR Gene) following 6 h purine starvation were determined in parallel from aliquots of the same culture. All qRT-PCR data were normalized to *UMPS*. The mean and standard deviation determined from two independent biological replicates is shown for each analysis.

## Discussion

### Proteome Analyses Offer Insight into Metabolome Reconfiguration during Purine Stress

We undertook a large-scale global proteome profiling experiment to identify how *L. donovani* promastigotes reorganize their proteome to deal with purine stress, a type of nutrient stress. The label-free approach of AMT tag is highly suited to the analysis of multiple biological replicates [47], which enabled even subtle abundance changes between purine-starved and purine-replete cells to be detected reproducibly and with a high degree of confidence. It is noteworthy that for those proteins where fold changes had previously been measured by western analyses [8] (i.e., for the purine permeases and various phosphoribosyltransferases), or by spectral counting in a separate comparative shotgun proteomics experiment (see Table S2), the comparative AMT tag data was in good agreement, emphasizing the accuracy of this approach in predicting even small protein abundance changes. Changes within the parasite proteome followed a chronological order with the first response primarily focused on enhancing purine acquisition at the cell surface, but this was succeeded by a broader transformation of the parasite proteome upon prolonged purine deprivation. With chronic purine stress, progressively more alterations within the parasite proteome were apparent. Indeed, by 48 h, approximately one-third of the proteins that could be detected by the AMT tag method within the parasite proteome were significantly changed in abundance. The challenge of future explorations will be to identify changes induced upon purine stress for those lower abundance proteins not easily detected by current methods used to profile the whole cellular proteome. Nevertheless, the current results have provided significant insight into proteome changes, and in some cases metabolic alterations, within purine-starved cells, and by extrapolating these findings to the entire annotated parasite proteome it is clear that the adaptive response to purine stress is both extensive and multifaceted in nature.

Remarkably, despite being growth-arrested, the metabolism of purine-starved cells appears to remain robust. Measurement of the reduction of resazurin to resorufin, a reaction which occurs primarily in the mitochondria of most cells and is essentially a measure of aerobic respiration and mitochondrial metabolism [103]–[105], indicated that even cells purine-starved for two weeks were essentially as metabolically active as those continuously grown in purine (Fig. S6). Within the 48 h proteome, more than 78% of the proteins that were significantly changed in abundance were upregulated. In these chronically purine-deprived cells, the proteomics data, supported by the leucine incorporation data (Fig. 5) and our previous cell cycle data, which showed an arrest in growth in G_1_ phase [8], indicated that there was a decrease in energy-intensive biosynthetic pathways, such as DNA and protein synthesis, and the catabolism of proteins and nucleic acids into their precursor molecules appeared favored. This is likely a reflection of the differing metabolic needs of these non-dividing cells, which are unencumbered of the requirement to replicate nuclear and cellular material for proliferation. Instead, within these cells proteins involved in digestive processes were generally upregulated, and in addition to promoting proteome remodeling, these factors would likely serve to facilitate the disposal of spent and potentially damaged macromolecules that would normally be dispersed during cell segregation.

The response to prolonged purine stress is complex and the proteomics data revealed upregulation of numerous proteins involved in cellular activities spanning amino acid and lipid metabolism, cell redox response, intracellular trafficking, and protein interaction. One pathway that was abundantly represented in the altered proteome of purine-starved cells was that of central carbon metabolism. By both AMT tag analysis and spectral counting, participants in glycolysis, gluconeogenesis, the pentose phosphate pathway, and TCA cycle were all observed to be upregulated (Tables S1, S2, S3). Most of the changes were subtle (two-fold or less) and what (if any) impact these changes exert on flux through these pathways cannot, *a priori*, be predicted from the steady state levels revealed by the proteome data. However, it is conceivable that the increase observed in glycolytic and TCA cycle components in purine-starved cells serves to compensate for dwindling ATP levels, the reduction of which we have recently confirmed (Martin *et al.*, unpublished). On the other hand, the observed increase in the levels of the gluconeogenic enzymes, phosphoenolpyruvate carboxykinase (LinJ.27.1710), pyruvate diphosphate kinase (LinJ.11.1000), and fructose-1,6-bisphosphatase (LinJ.04.1170), could also signify a boost in the synthesis of glucose-6-phosphate from oxaloacetate or pyruvate, which, although resulting in a net loss of ATP currency in the cell, could also favor flux through the pentose phosphate pathway. (Although it should be noted that both phosphoenolpyruvate carboxykinase and pyruvate diphosphate kinase can also function in the opposing direction to regenerate glycosomal ATP levels and facilitate flux through the glycolytic pathway). The pentose phosphate pathway has been critically implicated in the response to oxidative stress in *Leishmania* and other organisms [106], [107]. Significantly, enzymes within the oxidative branch of the pathway that irreversibly catalyze the conversion of glucose-6-phosphate to ribulose-5-phosphate, providing a crucial mechanism for NADPH regeneration, were upregulated at the protein level, as were those enzymes responsible for the recycling of ribulose-5-phosphate back to glucose-6-phosphate (Fig. S1). The importance of cellular reductive energy in the form of NADPH in purine-starved cells may also be underscored by the fact that two other lesser-known mechanisms for generating NADPH, NADP-linked malic enzyme (LinJ.24.0780) and isocitrate dehydrogenase (LinJ.10.0310), were also upregulated at the protein level in these parasites. It is notable that the changes recorded for central carbon metabolism in *Leishmania* during purine stress have also been described in response to assorted stresses in a variety of other organisms including bacteria, protozoa, yeast, nematodes, and mammalian cells [107]–[111], underscoring the importance of these pathways in the adaptive mechanism to microenvironmental stress.

Much of the evidence points to purine-starved cells being under greater oxidative stress during purine starvation. Proteins associated with protein folding and quality control, such as heat shock proteins, peptidyl-prolyl cis-trans isomerases, and calreticulin (LinJ.31.2670) were all upregulated during purine stress, as were those proteins involved in cell redox homeostasis. Moreover, the antioxidant capacity of purine-starved cells was significantly and progressively improved in comparison to that of purine-replete cells (Figs. 6 and S2). In particular, enzymes involved in first-line oxidant defense such as the superoxide dismutases (LinJ.32.1910 and LinJ.08.0300) and ascorbate-dependent peroxidase (LinJ.34.0070) were all augmented at the protein level, as were those enzymes γ-glutamylcysteine synthetase (LinJ.18.1660), ornithine decarboxylase (LinJ.12.0100), and spermidine synthase (LinJ.04.0570) that participate in the biosynthesis of trypanothione, a low molecular weight dithiol that acts as a glutathione surrogate in these parasites [112], [113]. Most significantly, trypanothione reductase (LinJ.05.0350), an NADPH-linked activity solely responsible for trypanothione reduction, was upregulated, along with tryparedoxin (LinJ.29.1250), a glutaredoxin-like protein, and peroxiredoxin (LinJ.29.1250), which are all key constituents of trypanothione redox metabolism [114]. Recently, the upregulation of these proteins, along with components of the pentose phosphate pathway and gluconeogenesis, was also implicated in the response to oxidative and nitrosative stress in *L. donovani* [115], suggesting that common stress response pathways are likely enacted regardless of the nature of the particular environmental stress.

Even though the majority of the proteome changes detected were modest, involving changes of two-fold or less (Fig. 1), many could be grouped to either the same metabolic pathway or were similar in their molecular function (Figs. 4, S1, S3, S4 and Table S3), denoting, perhaps, a significant role for these biological processes in acclimatization to purine stress. One pathway that exhibited multiple, but small changes at the protein level was that of proline biosynthesis and interconversion, where the components of the pathway were increased or decreased by three-fold or less in a manner predicted to favor proline accumulation in purine-starved parasites (Fig. S3 and Tables S1 and S3). Upon examination of intracellular free proline levels in purine-starved *versus* purine-replete cells, a significant increase at both 24 and 48 h was observed (Fig. 7). While these data support our proteomics findings, they also provide compelling evidence that small changes in the levels of multiple proteins within the same pathway can lead to significant metabolic alterations. Thus, we conjecture that the effect of these cumulative, subtle changes in multiple pathways might confer a metabolic flexibility on purine-starved parasites, enabling their rapid adaptation and response to fluctuations within the host nutritional and physiological environment.

### Proteome Restructuring Is Achieved by a Complex Assortment of Mechanisms

In addition to identifying the adaptive changes in the purine-starved cell proteome, it was also important to investigate the molecular mechanisms underlying proteome restructuring. Canonical regulation of the transcriptome through promoters and transcriptional enhancers is absent in these parasites, and instead, kinetoplastids rely on post-transcriptional regulatory mechanisms, many of which are still being defined within these parasites [36], [37], [116]. To survey the changes in the parasite global transcriptome upon the induction of purine stress, we used the powerful method of RNA-seq to measure the fold changes at the mRNA level and compared them with those quantified at the protein level. Significantly, there was a high degree of discordance between the mRNA and protein abundance data, and the results signified the complexity of proteome remodeling that is realized by a diverse assortment of mechanisms at the mRNA, translational, and post-translational level. For a select group of candidates, the role of 5′ and 3′ UTRs in directing changes produced at the translational level were distinguished from those at the mRNA level using a Dual-Luciferase reporter strategy, where one copy of the candidate gene was replaced with a *Fluc* reporter. While regulatory mechanisms could be ascribed for many of the candidates, it was also apparent that their regulation is complex and is achieved at multiple post-transcriptional levels. For example, the purine permeases LdNT1 and LdNT3 showed regulation at both the mRNA and translational level, while LdNT2, another purine transporter, was only regulated translationally. In the case of LdNT2, our previous work demonstrated that expression of GFP-tagged LdNT2 from an episome lacking the cognate *LdNT2* UTRs was upregulated 10-fold in response to purine starvation for 72 h, suggesting additional mechanisms involving post-translational stabilization of LdNT2 protein or perhaps a regulatory role for the *LdNT2* CDS [8]. The complexity of post-transcriptional regulation invoked during purine starvation is also illustrated by the differing responses for those mRNAs arising from various *Fluc* integrations (Table 2), where the fold changes in mRNA abundance were consistently lower than those for the corresponding wild type allele, with the exception of LdNT4, which is not upregulated after 24 h of purine starvation. Thus, it is likely that both the UTRs and the CDS of those purine-responsive candidates described in Table 2 contribute to regulation. Indeed, modulation of mRNA levels by *cis*-acting regulatory elements encoded within various CDSs has been demonstrated in the related kinetoplastids *T. brucei* [117] and *T. cruzi* [118], and for numerous mammalian mRNAs [119]. The discrepancy in mRNA abundance changes between *Fluc* and the corresponding allele may also be a reflection of the differences in the intracellular location of mRNA translation. Endoplasmic reticulum association has been shown to increase mRNA stability [120] and, under conditions of cellular stress, protect mRNAs from incorporation into stress granules, thus preserving their translational capacity [121], and potentially protecting the mRNA from degradation through association with processing bodies or P-bodies. All but one of the genes, that for oxidoreductase, examined *via* the Dual-Luciferase System (Table 2) encode membrane proteins whose obligatory translation at the ER could facilitate mRNA stabilization. In contrast, mRNAs from alleles in which a membrane protein CDS was replaced by *Fluc* (which encodes for a soluble protein lacking transmembrane domains) are likely translated in the cytosol and may not have access to ER-associated factors that protect specific mRNAs from the general decrease in cellular RNA content observed in response to purine stress [8].

In summary, it is highly probable that more complex regulatory mechanisms than could be uncovered here contribute to the dynamic changes in the proteome, including changes in mRNA subcellular location (*e.g.* stress granules, P-bodies, or ER association) that alter interactions with the translation machinery and other *trans*-acting factors, and post-translation modifications that enhance or reduce protein stability. A broader survey of the post-transcriptional regulatory mechanisms for the other candidates identified in our proteomic screen will likely illuminate some of these mechanisms by identifying common regulatory *cis*-acting sequences and *trans*-acting factors involved in the coordinated and temporal response to purine stress.

### Purine Stress and Parasite Differentiation

Previously, both nutrient depletion and environmental stressors such as decreased pH, increased temperature, and exposure to antimonial drugs have been observed to trigger parasite differentiation [18], [122]–[124], and recently an intriguing link between adenosine depletion and the induction of metacyclogenesis has also been proposed [15]. Although we cannot rule out the possibility that some of the temporal proteome changes that we detected upon purine stress are due to the induction of parasite differentiation, it seems highly unlikely that the remodeling of those pathways involved in purine capture, salvage, and interconversion, is due to parasite differentiation. Evaluation of those mRNAs known to be increased in metacyclic and differentiating parasites by SL RNA-seq (Table S5) or by qRT-PCR (Table 1), such as *SHERP* (LinJ.23.1210, LinJ.23.1230 and refs. [124], [125]), *A2* (LinJ.22.0670 and refs. [122], [126]), and *META1* (LinJ.17.0990 and ref. [127]), exhibited only a minor increase for *SHERP* (Table 1 and Table S5) and even a decrease for *A2* (Table S5), although the *META1* mRNA by SL RNA-seq showed an increase of ∼2-fold (Table S5). Purine stress appears to induce a pronounced elongation of the *L. donovani* cell body [8] that is distinctly different than the morphological changes ascribed to metacyclic parasites [128], although the morphology is similar to that described for the nectomonad stage, one of the earliest differentiating stages within the anterior abdominal mid-gut of the sand fly [129]. Thus, it is not inconceivable that some of the stress response pathways triggered during purine starvation are also induced during the early stages of parasite differentiation. Indeed, some of the changes chronicled in this manuscript have also been described during the early stages of differentiation in culture when late log phase *L. donovani* promastigotes were induced to convert to amastigotes, the mammalian infectious life cycle form, by temperature and pH shift of the culture conditions [86], [122].

In summary, this manuscript provides a detailed examination of *L. donovani* proteome dynamics upon the induction of purine starvation, a type of nutrient stress. Moreover, the data revealed that a complex assortment of molecular mechanisms conspire to induce an extensive reprogramming of the parasite proteome, which in some cases could be extrapolated to the parasite metabolome, and enable parasite survival even under conditions of extreme purine scarcity. In large part, this is achieved by the rebalancing of intracellular ATP expenditure, signified by the apparent decrease in energy-intensive processes such as genome replication and protein synthesis. Remarkably, despite being growth-arrested, purine-starved cells remained metabolically active, augmenting several pathways, many of which likely are part of a common stress response mechanism in these parasites [86], [115].

## Materials and Methods

### Cell Lines and Cultivation

The visceralizing *L. donovani* strain 1S2D [130] clonal derivative LdBob [131] was employed in these studies. Wild type LdBob promastigotes were routinely cultured at 26°C in 5% CO_2_ in modified Dulbecco's Modified Eagle-Leishmania (**DME-L**) medium [132] that lacked bovine serum albumin and was supplemented with 5% serum plus (Sigma-Aldrich), 1 mM glutamine, 1X RPMI 1640 vitamin mix, 10 µM folate, hemin (2 ml from a 500X stock containing 2.5 g l^−1^), and 100 µM hypoxanthine as a purine source. To elicit purine starvation conditions, cells were grown in DME-L lacking hypoxanthine but with all other media components present.

### Treatment of Cells for Downstream Proteomic Analyses

Logarithmically growing cells were seeded at 2×10^6^ cells ml^−1^ in 5 roller bottles corresponding to 5 individual biological replicates and grown for 24 h prior to the start of purine starvation in complete DME-L supplemented with 100 µM hypoxanthine. At t = 0 h, 2×10^8^ cells from each replicate were processed for downstream proteomic analyses as detailed below. The remaining cells in each replicate were prepared in parallel as described. Briefly, cells were washed twice in DME-L medium lacking purines and resuspended at 2×10^6^ cells ml^−1^ in either purine-replete media (DME-L supplemented with 100 µM hypoxanthine) or in purine-deplete media (DME-L lacking hypoxanthine). At time points corresponding to 6, 12, 24, and 48 h after the initiation of purine starvation, 2×10^8^ cells were removed from both the purine-replete and purine-starved cultures and processed for downstream proteomic analyses as described below. Note that the densities for the cells growing in purine-replete media were adjusted every 24 h to ensure that they remained in the log phase of growth and thus, any differences identified between the proteomes of purine-starved and purine-replete cells were due solely to the effects of purine depletion.

### Sample Preparation for Proteomic Analyses

The reserved cells for proteomic analyses, 45 samples in total, consisting of 5 biological replicates per condition (purine-starved or purine-replete) and at time points of 0 h (purine-replete only) and 6, 12, 24, and 48 h (purine-starved and purine-replete), were washed twice in 50 ml of Dulbecco's Phosphate Buffered Saline (D-PBS) to remove media contaminants, resuspended in 500 µl of D-PBS, and transferred to a microcentrifuge tube. Cells were pelleted at 1500× *g*, the supernatant removed and the cell pellets put on ice. Each cell pellet was resuspended in 500 µl of complete lysis buffer (100 mM ammonium carbonate pH 7.8 containing 8 M urea) and incubated on ice for 10 min. After 10 min of incubation, a small aliquot of the cell lysate was reserved for analysis with the Micro BCA Protein Assay kit (Thermo Scientific) to determine protein concentration, and the rest of the lysate flash-frozen in liquid N_2_ and stored at −80°C until ready for downstream proteomic analysis.

### Protein Extraction and Digestion

For each of the 45 samples, 300 µg of protein extract was reduced with 5 mM dithiothreitol for 1 h at 37°C, and subsequently, alkylated with 15 mM iodoacetamide (**IAA**) for 2 h at 25°C in the dark. Each sample was then diluted 10-fold with 50 mM NH_4_HCO_3_ (pH 8) containing 1 mM CaCl_2_ and digested for 3 h at 37°C with trypsin, added at a 1∶50 trypsin to protein ratio. Samples were acidified with trifluoroacetic acid prior to desalting with a Discovery-C18 SPE cartridge (SUPELCO). Aliquots of peptides (20 µg) from each of the purine-replete or the purine-starved digests were pooled into two samples (purine-replete versus purine-starved). Peptides from each pooled sample at a concentration of 350 µg were fractionated using a high pH (pH 10) reversed-phase liquid chromatography strategy, which has been previously described [133], and collected across 96 fractions, which were then concatenated into groups of 4 to yield 24 fractions. The fractions were dried in a Speed-Vac to remove the solvent and resuspended in 170 µl of Nanopure water.

### Reversed-phase Capillary LC-MS/MS and LC-MS Analyses

LC-MS/MS analysis of the pooled fractions described above was used to generate a reference database of peptide markers defined by accurate masses and elution times, i.e., AMT tag (described in the paragraph below and refs. [46]–[48]). The AMT tag database then served as a comprehensive “look up” table for the subsequent higher throughput LC-MS analyses described below. For the AMT tag database generation, each of the 24 fractions corresponding to the purine-replete and purine-starved cells were analyzed using a 4-column, custom-built, capillary LC system coupled online via an in-house manufactured electrospray ionization (**ESI**) interface to an LTQ-Orbitrap mass spectrometer (Thermo Scientific, San Jose, CA) [134]. To identify the eluting peptides, the LTQ-Orbitrap instrument was operated in a data-dependent MS/MS mode for the top six abundant precursor ions in each full MS scan. Following the AMT tag database generation, LC-MS analyses with full MS scan (400–2,000 *m/z* range) were performed on the 45 unfractionated purine-replete and purine-starved peptide samples described above to generate quantitative data. For this, samples were analyzed in random order using the same chromatographic and electrospray conditions as described for the LC-MS/MS analyses except that the LC system was interfaced to an Exactive Mass Spectrometer (Thermo Scientific).

### Development of an AMT Tag Database for *L. donovani*

MS/MS data were searched against a decoy protein database consisting of forward and reversed sequences entries extracted from a May 18, 2010 download from the Wellcome Trust Sanger Institute (http://www.sanger.ac.uk/) of a *Leishmania infantum* FASTA protein database (LinJwholegenome_20080508.v3.0a.fasta) using SEQUEST analysis software [135]. When searching, SEQUEST used a dynamic mass modification for methionine oxidation (15.9949 Da) and a static mass modification for IAA induced cysteine alkylation (57.0215 Da). Peptides with an estimated false discovery rate <0.1% based on a target-decoy database search [136] were stored as AMT tags in a Microsoft SQL Server database. The elution times for these peptides were normalized to a range of 0 to 1 using a predictive peptide LC normalized elution time (**NET**) model and linear regression, as previously reported [137].

### AMT Tag Identification of Tryptic Peptides from Purine-Replete and Purine-Starved Cells

Identification and quantification of the detected peptide peaks were performed utilizing the AMT tag approach [46]–[48]. Briefly, multiple in-house developed, but publicly available, informatics tools were used to process LC-MS data and correlate the resulting LC-MS features to build an AMT tag database containing accurate mass and LC separation elution time information for peptide tags generated from *Leishmania donovani* proteins. Among the tools used were algorithms for peak-picking and for determining isotopic distributions and charge states [138]. Further downstream data analysis incorporated all of the possible detected peptides into a visualization program VIPER [139] to correlate LC-MS features to the peptide identifications in the AMT tag database. The VIPER results were matched with a refined mass tolerance of ±2 ppm and a refined NET tolerance of ±2%. VIPER provided an intensity report for all detected features, normalized LC elution times via alignment to the database, and feature identification. In DAnTE software [140], peptide peak intensity values were converted to a log_2_ scale and proteins with ≥2 peptides (Rrollup parameters) were statistically compared between the two conditions (plus or minus purine) at each time point utilizing ANOVA performed as a t-test (with a minimum of 3 data points per condition).

### Gene Ontology Classifications

UniProt identifiers for all of those proteins with significantly altered abundance (p-value of ≤0.05 and log_2_ value of ≥ 0.5 or ≤−0.5) were submitted to the AgBase server to assign a Gene Ontology (**GO**) classification (http://www.agbase.msstate.edu and ref. [141]). All proteins were then grouped according to their GO category, or, alternatively, based upon predicted or known molecular function based upon searches of the UniProt database (http://www.uniprot.org/) and ref. [142]), PubMed (http://www.ncbi.nlm.nih.gov/pubmed/), or by using the BLAST algorithm (http://blast.ncbi.nlm.nih.gov/Blast.cgi and ref. [143]). In those cases where neither a GO category nor molecular function could be ascribed, proteins were classified as of “unknown function”.

### Radiolabeled Leucine and Uracil Incorporation Assays

Logarithmic cells, either continuously grown in 100 µM hypoxanthine or starved for purine for 24 h, were washed twice in growth media (**DMEL-Bob-Leu^−^**) that lacked both leucine and uracil and supplemented with (purine-replete cells) or without (purine-starved cells) 100 µM hypoxanthine. Cells were resuspended in DMEL-Bob-Leu^−^ plus or minus 100 µM hypoxanthine at a density of 5×10^7^ cells/ml to which 1 µCi ml^−1^ of [4,5-^3^H]-leucine (Sp. Act. 144 Ci mmol^−1^) and 0.1 µCi ml^−1^ of [2-^14^C]-uracil (Sp. Act. 57 mCi mmol^−1^) was added. At t = 20, 40, 60, and 80 min, 5×10^7^ cells from the purine-replete and purine-starved cultures were washed twice in ice-cold PBS, resuspended in 0.5 ml of ice-cold PBS to which an equal volume of a 20% trichloroacetic acid/water mix was added, and precipitated on ice for 60 min. The acid-precipitated material was collected onto glass fiber filters *via* vacuum manifold, washed twice with 1 ml of a 10% trichloroacetic acid/water mix, followed by two washes with 1 ml of ice-cold ethanol. The filters were dried and radioactivity incorporated into the trichloroacetic acid-precipitated pool quantitated by liquid scintillation counting on a Beckman LS6500 liquid scintillation counter.

### Quantitation of ROS in *Leishmania*

ROS measurements were performed based upon the published method of ref. [144]. Log phase promastigotes, either continuously grown in 100 µM hypoxanthine or starved for purine for 24–72 h, were washed twice in Hank's Balanced Salt Solution supplemented with 25 mM glucose (**HBSS-G**) and either plus (purine-replete cells) or minus (purine-starved cells) 100 µM hypoxanthine. Cells were seeded at a density of either 0.1, 0.5, or 1×10^8^ cells per well in HBSS-G plus or minus 100 µM hypoxanthine and containing 25 µg ml^−1^ of the cell-permeant fluorescein derivative 2′,7′-dichlorodihydrofluorescein diacetate (H_2_DCFDA) (Sigma-Aldrich) and incubated at 25°C for 30 min. Fluorescence was measured with a SpectraMax M2 Microplate Reader (MolecularDevices GmbH, Ismaning/München, Germany) at wavelengths of 485 nm for excitation and 535 nm for emission. Relative fluorescence units (**RFU**) measured were normalized to 10^6^ cells.

### Determination of Free Intracellular Proline in *Leishmania*

The free intracellular L-proline concentration was determined for purine-replete and purine-starved cells by the method of [145]. 10^8^ promastigotes were harvested at 24 and 48 h post induction of purine starvation. Cells were washed in PBS and proteins precipitated using 20% trichloroacetic acid in PBS for 30 min on ice. Lysates were centrifuged at 10,000× *g* for 30 min at 4°C. A 200 µl aliquot of the resultant supernatant was mixed *via* inversion with 200 µl of an acid ninhydrin solution (0.25 g ninhydrin, 6 ml of glacial acetic acid, and 4 ml of 6 M phosphoric acid) and 200 µl of glacial acetic acid, and incubated at 100°C for 1 h. A standard curve for L-proline (0–500 µM) was prepared in parallel. All reactions were stopped by chilling of the samples on ice and the chromagen extracted into 400 µl of toluene. For enumeration, a 100 µl aliquot of the toluene solution was measured in a quartz microcuvette at 520 nm using a Beckman DU-600 spectrophotometer.

### Whole Transcriptome Shotgun Sequencing (RNA-Seq)

Early log phase *L. donovani* promastigotes were cultivated in purine-replete and purine-deplete media for 24 h as described above for the AMT tag analyses, after which time 1×10^8^ cells were removed from each condition, harvested by centrifugation and washed twice in D-PBS to remove all media components. Total RNA from the purine-replete and purine-deplete cells was isolated using an RNeasy kit (Qiagen Inc., Valencia, CA) according to the manufacturer's protocol. Next Generation Sequencing (**NGS**) libraries were constructed by priming ∼1 µg of total RNA from either the purine-starved or purine-replete sample with a random hexamer primer (TCCGATCTCTNNNNNNN) for first strand cDNA synthesis and an oligonucleotide primer (TCAGTTTCTGTA) derived from the *Leishmania* splice leader (**SL**) sequence for second-strand cDNA synthesis. This strategy is effective in identifying the 5′ end of individual mRNAs, as well as differences in splice site usage [94]. The resultant cDNA was amplified by PCR for 12–15 cycles using the oligonucleotides (CAAGCAGAAGACGGCATACGAGCTCTTCCGATCTCT and AATGATACGGCGACCACCGACACTCTTTCCCTACATCAGTTTCTGTACTTTA) that overlapped with the initial primers and containing adapters for subsequent cluster generation and sequencing using Solexa sequencing by synthesis (**SBS**) technology. Short-read (36-nt) sequences were obtained using a SL-specific sequencing primer (CACTCTTTCCCTACATCAGTTTCTGTACTTTA) on the Genome Analyzer IIx platform at the University of Washington High Throughput Genomics Unit. The FASTQ sequence data files were analyzed at Seattle BioMed using a customized NGS analysis pipeline. After quality checking, the reads were aligned with the *L. donovani* genome using Bowtie [146], and the results uploaded into a relational database. These analyses identified both the major and alternative splice sites for each gene, as well as the number of reads at each site. The latter was used to determine the expression level of each mRNA. To normalize the purine-starved and purine-replete samples for reads/mRNA comparison, read numbers were divided by the median number of reads per mRNA in each sample [147].

### qRT-PCR Analyses

Total cellular RNA was isolated from 2×10^8^ *L. donovani* promastigotes using an RNeasy Mini Kit (Qiagen Inc., Valencia, CA) following the manufacturer's protocol. All additional qRT-PCR reagents were from Life Technologies (Grand Island, NY). To minimize potential contamination by genomic DNA, the purified RNA was subjected to DNaseI digestion using the Turbo DNA-*free* kit. First-strand cDNA was synthesized with a High Capacity cDNA Reverse Transcription kit using 2 µg of total RNA as per the manufacturer's instructions. For qRT-PCR, 5 µl of diluted first-strand cDNA reaction corresponding to 20 ng of input RNA was included in a 20 µl reaction with 5 pmol primers (see Table S6) and 10 µl 2X SYBR Select Master Mix. The reactions were run on an Applied Biosystems 7500 Real-Time PCR System under the following conditions: 50°C for 2 min and 95°C for 10 min, followed by 40 cycles of 95°C for 15 s and 60°C for 1 min. A final thermal dissociation analysis was performed for each reaction to confirm that the PCR generated a single amplification product. The relative abundance of target amplicons between purine replete and starved samples was determined via the 2^−ΔΔC^_T_ method [148].

### Dual-Luciferase Assay

Constructs containing the *Photinus pyralis* (firefly) luciferase gene fused at the 3′ end to the blasticidin-S deaminase gene (GenBank accession number KF035118; designated as ***Fluc*** in the text and Tables 2 and 3) and flanked by ∼1 kb of 5′ and 3′ sequence adjacent to the coding sequence of the corresponding genes of interest were assembled by the method of multifragment ligation [149]. (See Table S7 for details about the primer sequences used for the amplification of the 5′ and 3′ fragments). These constructs were linearized with *PacI* and transfected using standard transfection techniques [131] into a recipient cell line in which one copy of *UMPS* [102] had previously been replaced by the *Renilla reniformis* luciferase gene fused at the 3′ end to the puromycin acetyltransferase gene (GenBank accession number KF035116; designated as ***Rluc*** in the text and legend of Tables 2 and 3). *Rluc* integrated at the *UMPS* locus served as a control for the Fluc reporter assays in both purine-replete and purine-starved cells, since we have previously established by western analyses and qRT-PCR [8], as well as by the proteomic and SL RNA-seq analyses described in this manuscript, that the abundance of *UMPS* mRNA and its corresponding protein does not alter during 48 h of purine starvation. Changes in *Fluc* activity arising from integration at various loci was measured at 6 and 24 h post induction of purine starvation and compared to the internal normalizer of *Rluc* using the Dual-Luciferase Reporter Assay System (Promega, Madison, WI). Briefly, 1 ml of purine-replete or purine-starved cells (∼2×10^6^ cells) was harvested by centrifugation at 16,000× *g* for 1 min at ambient temperature and washed in 1 ml of PBS. Cells were resuspended in 1 ml of 1X Passive Lysis Buffer provided with the Dual-Luciferase Reporter Assay System kit, vortexed briefly, and then subjected to end-over-end rotation at ambient temperature for 20 min to achieve complete lysis. Twenty µl of the cell lysate was transferred to a well in a black flat-bottom 96-well assay plate (Corning, Amsterdam) and assayed according to the Dual-Luciferase Reporter Assay System protocol using a Veritas Microplate Luminometer (Turner BioSystems, Sunnyvale, CA). For each cell lysate, the relative light units (**RLUs**) arising from Fluc was divided by the RLUs arising from Rluc to obtain a normalized value, and the fold change in purine-starved cells calculated by dividing the normalized value with that from the corresponding purine-replete sample. Note that details of the construction of *Fluc* and *Rluc* will be reported elsewhere (Soysa *et al.*, unpublished).

## Supporting Information

Figure S1 **A schematic of the changes in the pentose phosphate pathway upon purine starvation.** Thick red arrows indicate those steps catalyzed by proteins (in red) that are upregulated and black arrows indicate those steps catalyzed by activities that are unchanged during purine starvation. The conversion of NADP^+^ to NADPH is represented by the blue arrows. Proteins marked by an * were absent from the 6–48 h proteome datasets. TriTrypDB accession numbers are given for each protein. *Abbreviations:* G6PDH, glucose-6-phosphate dehydrogenase; 6-PGL, 6-phosphogluconolactonase; 6-PGDH, 6-phosphogluconate dehydrogenase; R5P Isomerase, putative ribose-5-phosphate isomerase; R5P Epimerase, putative ribulose-5-phosphate-4-epimerase; PRPPS, phosphoribosyl pyrophosphate synthetase; G6P Isomerase, glucose-6-phosphate isomerase.
(TIF)

Figure S2 **Response of purine-starved and purine-replete parasites to H_2_O_2_.** Purine-replete (open bars) and purine starved (24, 48 h, light and dark grey bars, respectively) promastigotes were exposed to 4 and 2 mM H_2_O_2_ as well as 5 µM of the ROS-generating compound menadione. Generation of ROS was measured by incubating parasites with the cell-permeant fluorescein derivative H_2_DCFDA. Error bars indicate standard deviation; data represent three independent biological replicates.
(TIF)

Figure S3 **A schematic of the changes in proline and glutamate metabolism upon purine starvation.** Thick red arrows indicate those steps catalyzed by proteins (in red) that are upregulated, the thin green arrow represents the step catalyzed by ProDH (in green) that is downregulated, and the black arrows indicate those steps that are unchanged during purine starvation. γGPR* could not be detected in the 6–48 h proteome datasets. TriTrypDB accession numbers are given for each protein. *Abbreviations:* P5CR, pyrroline-5-carboxylate reductase, putative; ProDH, proline dehydrogenase, putative; P5CDH, delta-1-pyrroline-5-carboxylate dehydrogenase, putative; P5CS, pyrroline-5-carboxylate synthetase-like protein; GK, putative glutamate 5-kinase; γGPR, putative γ-glutamyl phosphate reductase; GDH, glutamate dehydrogenase; GS, putative glutamine synthetase.
(TIF)

Figure S4 **A schematic of the changes in sphingoid base and phospholipid metabolism upon purine starvation.** Thick red arrows indicate those steps catalyzed by proteins (in red) that are upregulated, green arrows indicate those steps catalyzed by proteins (in green) that are downregulated, and black arrows indicate those steps catalyzed by activities that are unchanged during purine starvation. Proteins marked by an * were absent from the 6–48 h proteome datasets. TriTrypDB accession numbers are given for each protein. *Abbreviations:* SPT, serinepalmitoyltransferase-like protein; PAP, phosphatidic acid phosphatase; SPL, putative sphingosine 1-phosphate lyase; ECT, putative ethanolamine-phosphate cytidylyltransferase; CEPT, putative choline/ethanolamine phosphotransferase; PEMT1/2, phosphatidylethanolamine-methyltransferase-like proteins 1 and 2; DAGK, diacylglycerol kinase-like protein.
(TIF)

Figure S5 **A comparison of the fold changes at the protein level with those at the mRNA level for various purine pathway activities.** For purine-starved cells the fold changes at the protein level at 24 h (closed circles) and 48 h (grey circles) were divided by the fold change at the mRNA level as measured by RNA-seq. Black dotted line represents an exact correlation between the fold changes at the protein and mRNA level, and the dashed lines a 4-fold difference between the protein and mRNA levels either up (red) or down (green). See the legend of Fig. 2 for a list of the abbreviations.
(TIF)

Figure S6 **Rates of resazurin reduction by purine-starved and purine-replete parasites.** Generation of fluorescence arising from the irreversible reduction of resazurin to the fluorescent product resorufin was measured over time for purine-replete cells (open circles), 24 h purine-starved cells (light grey circles), 48 h purine-starved cells (dark grey circles), and cells starved for purine for two weeks (closed circles). Error bars indicate standard deviation, data represent two biological replicates for purine-replete cells and 3 biological replicates for all sets of purine-starved cells.
(TIF)

Table S1 **Accurate mass and time tag proteomics data derived from purine-starved and purine-replete cells.** The tabulated AMT tag data for all proteins identified at 6, 12, 24, and 48 h can be found under the ‘Protein Summary’ tabs; proteins significantly changed (p-value <0.05) are described under the ‘Significantly Changed’ tabs; and peptide matching information under the tabs ‘Relative Peptide Abundance’ and ‘Percent Coverage of Proteins’. Accession numbers correspond to the annotated *L. infantum* genome in TriTrypDB version 4.0.
(XLSX)

Table S2 **Summary of the spectral counting data derived from purine-starved and purine-replete cells at 24 h.** The data for all proteins identified by spectral counting can be found under the ‘Proteome’ tab and peptide information under the ‘Peptide Summary’ tab. Analysis of the spectral counting distributions between purine-starved and purine-replete cells can be found under the ‘Quant_Analysis’ tab (see Text S1 for an explanation of the data analyses). A comparison of the AMT tag and spectral counting data for those candidates significantly changed at 24 h by the AMT tag method is given under the ‘AMT vs. SpC Data at 24 h’ tab. Accession numbers correspond to the annotated *L. infantum* genome in TriTrypDB version 4.0.
(XLS)

Table S3 **Predicted gene ontology and biological function for proteins altered in response to purine starvation.** Proteins significantly altered (p-value of ≤0.05) by a log_2_ abundance ratio of either ≥0.5 or ≤−0.5 in purine-starved parasites are classified in terms of their biological and metabolic functions (see *Material and Methods* for the analysis methods).
(XLSX)

Table S4 **Summary of proteomics data for predicted glycosomal proteins.** The *L. infantum* genome was searched *via* the TriTrypDB interface for proteins containing a peroxisomal targeting signal (**PTS**) at either the C-terminus (PTS1) or N-terminus (PTS2), using the criteria described in ref. [77]. Only candidates with protein abundance data from the AMT tag analyses are listed. Those proteins with a log_2_ abundance ratio of ≤−0.5 are highlighted in green, and ≥0.5 in pink. Accession numbers correspond to the annotated *L. infantum* genome in TriTrypDB version 4.0.
(XLS)

Table S5 **Purine-starved and purine-replete SL RNA-seq data at 24 h.** The raw number of reads mapped to each gene for the purine-replete (ES001) and purine-starved (ES002) libraries are depicted. Accession numbers correspond to the annotated *L. infantum* genome in TriTrypDB version 4.0. Log_2__med refers to the log_2_ of the median-normalized ratio of reads between the purine-starved and purine-replete libraries.
(XLSX)

Table S6 **Comparison of fold changes at the protein and mRNA level in purine-starved cells.** The log_2_ abundance ratios for proteins at 24 h and 48 h were compared with the log_2_ expression ratio at 24 h for the corresponding mRNAs as measured by SL RNA-seq. Accession numbers correspond to the annotated *L. infantum* genome in TriTrypDB version 4.0.
(XLSX)

Table S7 **Primers for qRT-PCR analysis.** Sequences for those primers used for the qRT-PCR analyses described in Tables 1–3 are depicted. Accession numbers correspond to those from GenBank and the annotated *L. infantum* genome in TriTrypDB version 4.0.
(XLSX)

Table S8 **Primers for Fluc or Rluc reporter constructs.** Primer sequences used for the construction of *Fluc* or *Rluc* reporter constructs for integration at the indicated loci via homologous recombination are given. Sequences corresponding to 5′ or 3′ targeting sequences (TS) flanking the indicated gene are in plain text, while the SfiI restriction sites that facilitate the single-step assembly of the targeting construct are highlighted in boldface type. Accession numbers correspond to the annotated *L. infantum* genome in TriTrypDB version 4.0.
(XLSX)

Text S1 **Additional description of materials and methods.**
(DOCX)
