# Supplementary figures and images for: Metabolic Reprogramming during Purine Stress in the Protozoan Pathogen Leishmania donovani
Source: PLoS Pathog. 2014 Feb 27;10(2):e1003938. doi: 10.1371/journal.ppat.1003938 (PMC3937319; doi:10.1371/journal.ppat.1003938)

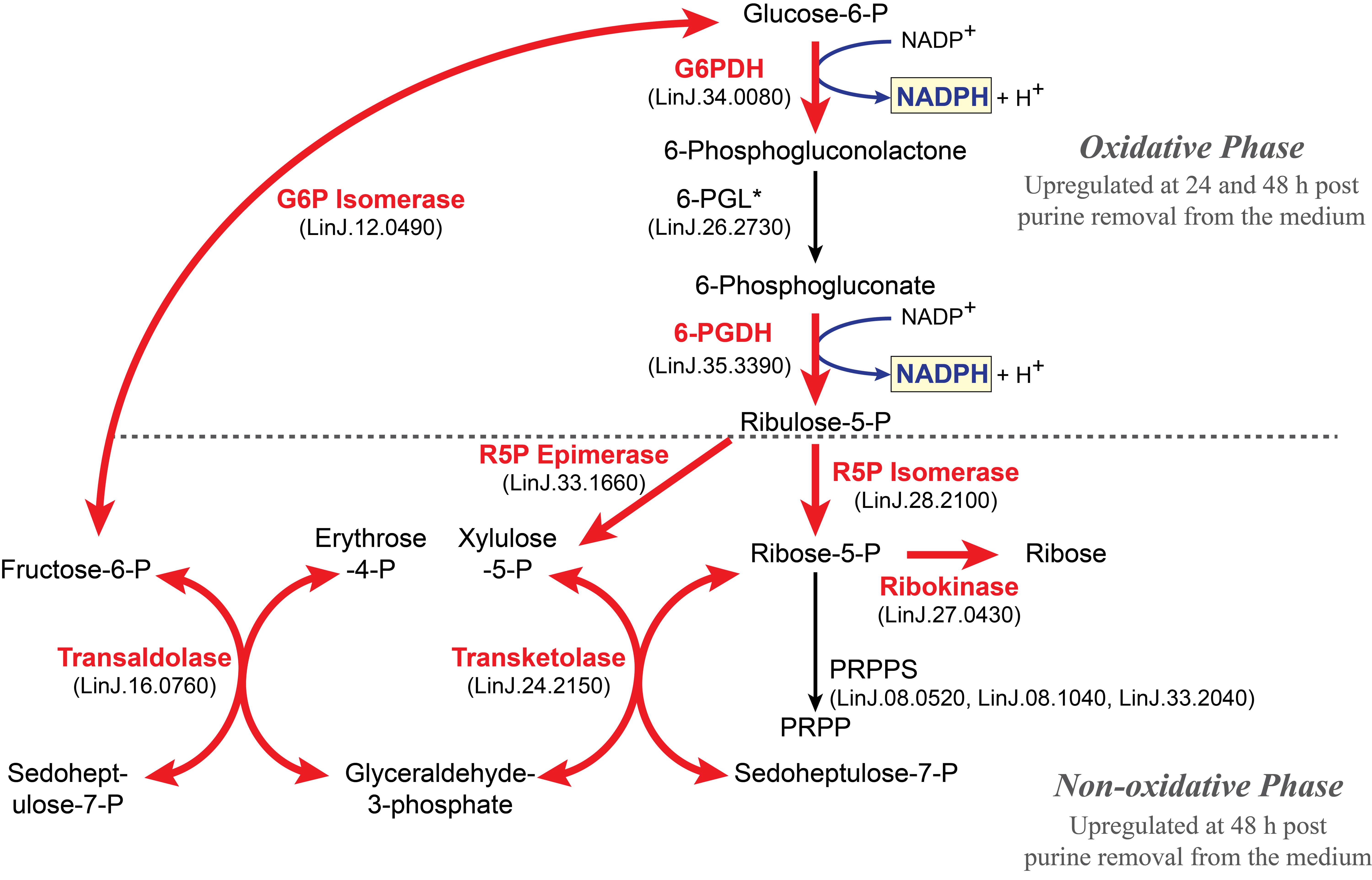

Supplement: Figure S1 — A schematic of the changes in the pentose phosphate pathway upon purine starvation. Thick red arrows indicate those steps catalyzed by proteins (in red) that are upregulated and black arrows indicate those steps catalyzed by activities that are unchanged during purine starvation. The conversion of NADP+ to NADPH is represented by the blue arrows. Proteins marked by an * were absent from the 6–48 h proteome datasets. TriTrypDB accession numbers are given for each protein. Abbreviations: G6PDH, glucose-6-phosphate dehydrogenase; 6-PGL, 6-phosphogluconolactonase; 6-PGDH, 6-phosphogluconate dehydrogenase; R5P Isomerase, putative ribose-5-phosphate isomerase; R5P Epimerase, putative ribulose-5-phosphate-4-epimerase; PRPPS, phosphoribosyl pyrophosphate synthetase; G6P Isomerase, glucose-6-phosphate isomerase. (TIF) [file ppat.1003938.s001.tif]

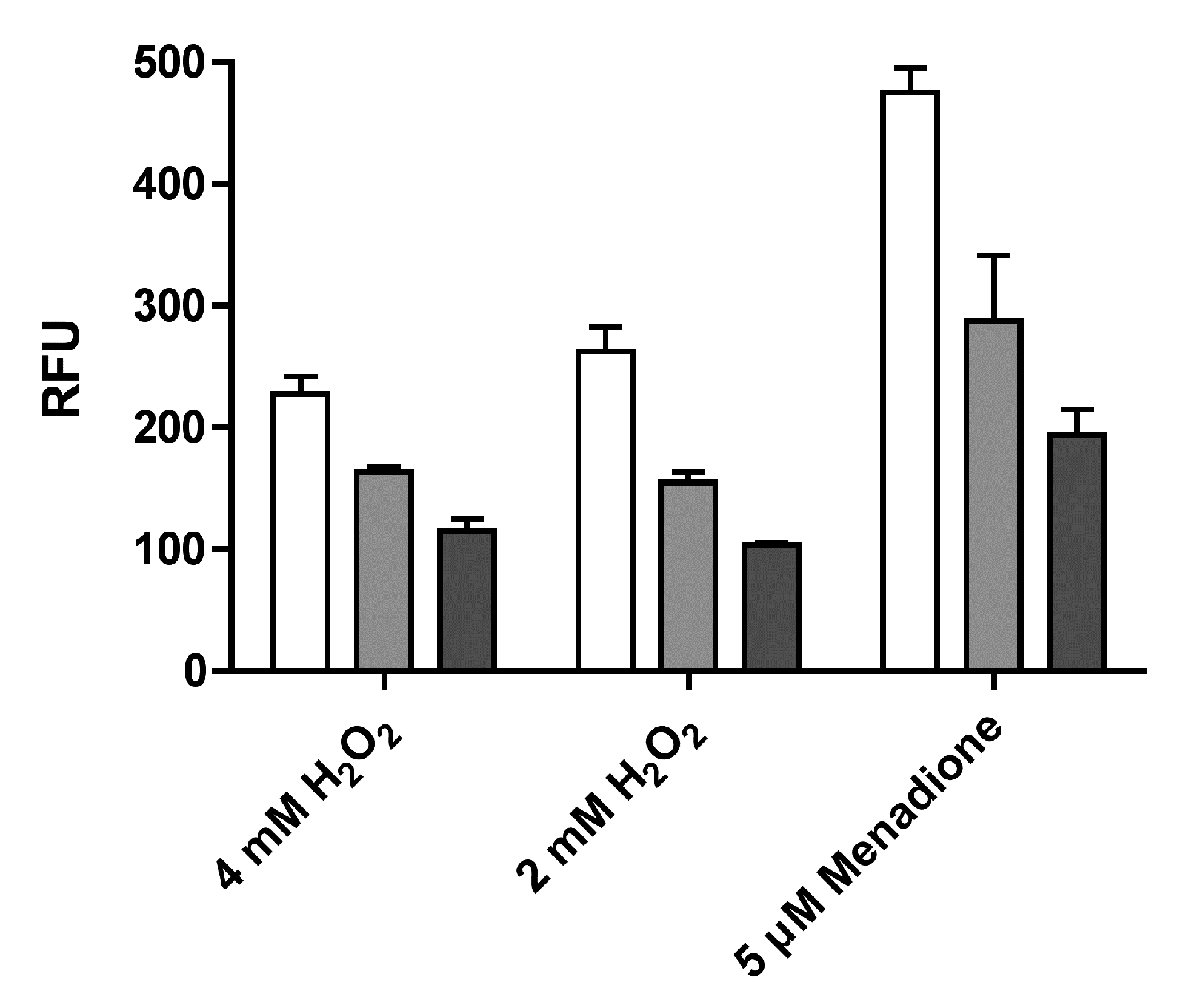

Supplement: Figure S2 — Response of purine-starved and purine-replete parasites to H2O2. Purine-replete (open bars) and purine starved (24, 48 h, light and dark grey bars, respectively) promastigotes were exposed to 4 and 2 mM H2O2 as well as 5 µM of the ROS-generating compound menadione. Generation of ROS was measured by incubating parasites with the cell-permeant fluorescein derivative H2DCFDA. Error bars indicate standard deviation; data represent three independent biological replicates. (TIF) [file ppat.1003938.s002.tif]

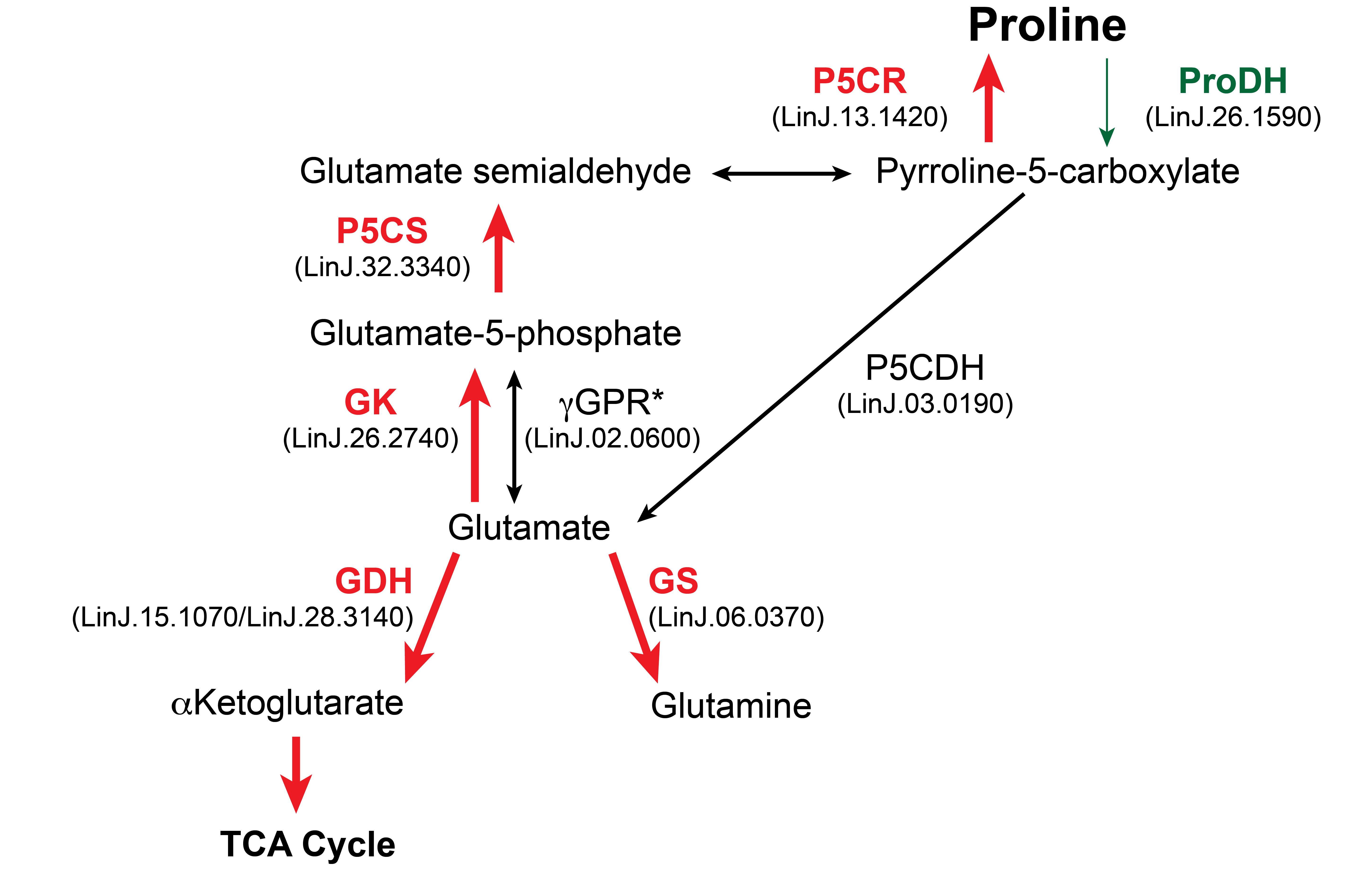

Supplement: Figure S3 — A schematic of the changes in proline and glutamate metabolism upon purine starvation. Thick red arrows indicate those steps catalyzed by proteins (in red) that are upregulated, the thin green arrow represents the step catalyzed by ProDH (in green) that is downregulated, and the black arrows indicate those steps that are unchanged during purine starvation. γGPR* could not be detected in the 6–48 h proteome datasets. TriTrypDB accession numbers are given for each protein. Abbreviations: P5CR, pyrroline-5-carboxylate reductase, putative; ProDH, proline dehydrogenase, putative; P5CDH, delta-1-pyrroline-5-carboxylate dehydrogenase, putative; P5CS, pyrroline-5-carboxylate synthetase-like protein; GK, putative glutamate 5-kinase; γGPR, putative γ-glutamyl phosphate reductase; GDH, glutamate dehydrogenase; GS, putative glutamine synthetase. (TIF) [file ppat.1003938.s003.tif]

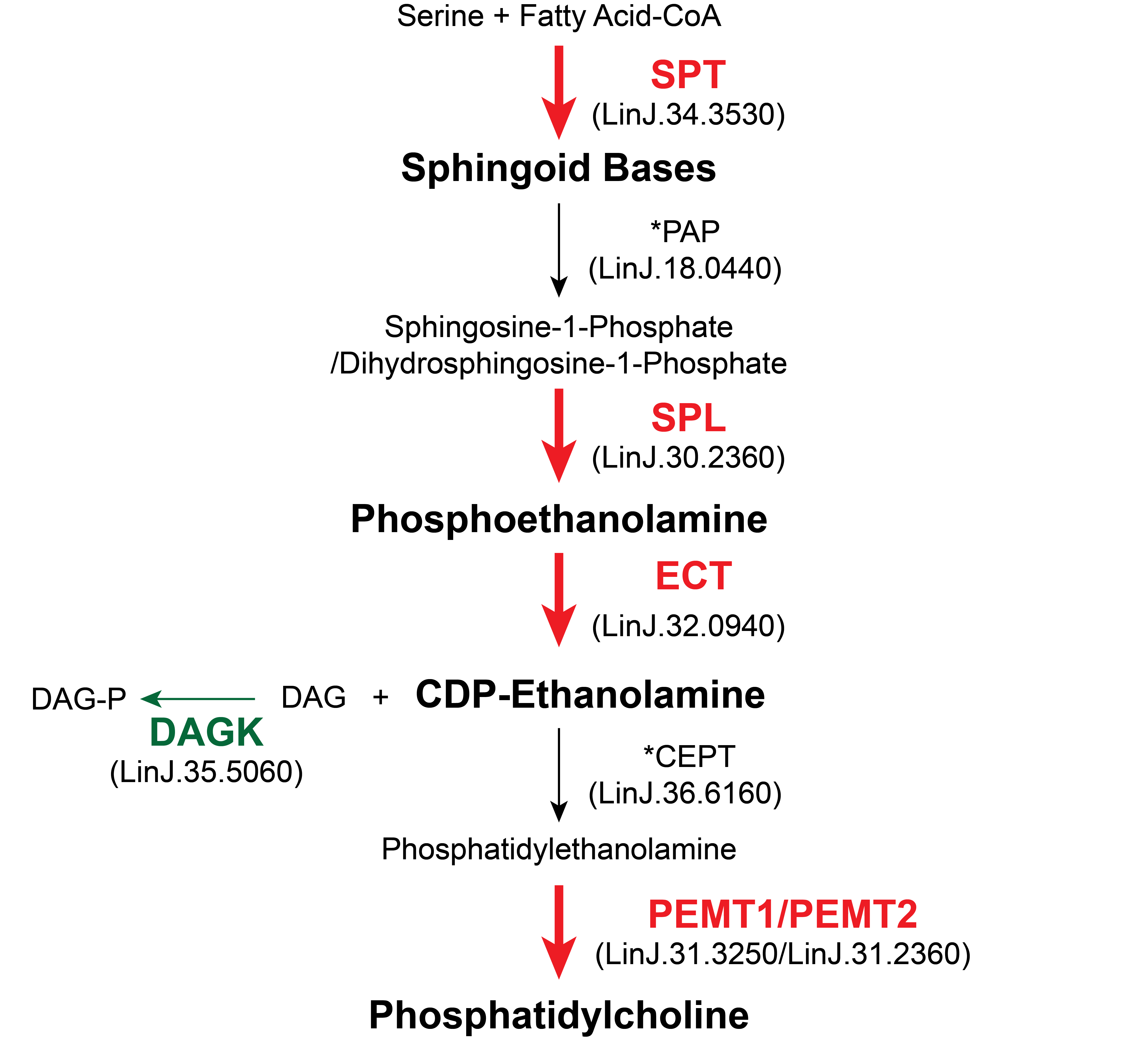

Supplement: Figure S4 — A schematic of the changes in sphingoid base and phospholipid metabolism upon purine starvation. Thick red arrows indicate those steps catalyzed by proteins (in red) that are upregulated, green arrows indicate those steps catalyzed by proteins (in green) that are downregulated, and black arrows indicate those steps catalyzed by activities that are unchanged during purine starvation. Proteins marked by an * were absent from the 6–48 h proteome datasets. TriTrypDB accession numbers are given for each protein. Abbreviations: SPT, serinepalmitoyltransferase-like protein; PAP, phosphatidic acid phosphatase; SPL, putative sphingosine 1-phosphate lyase; ECT, putative ethanolamine-phosphate cytidylyltransferase; CEPT, putative choline/ethanolamine phosphotransferase; PEMT1/2, phosphatidylethanolamine-methyltransferase-like proteins 1 and 2; DAGK, diacylglycerol kinase-like protein. (TIF) [file ppat.1003938.s004.tif]

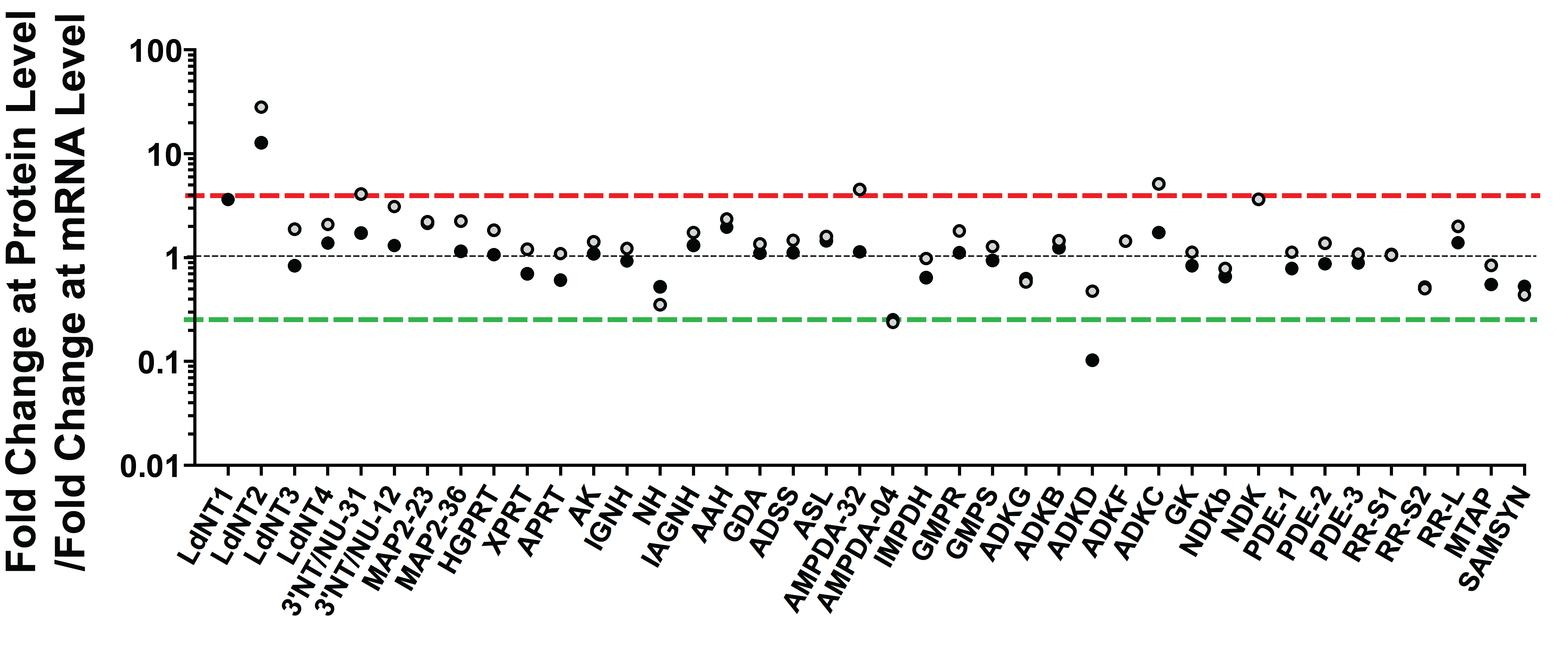

Supplement: Figure S5 — A comparison of the fold changes at the protein level with those at the mRNA level for various purine pathway activities. For purine-starved cells the fold changes at the protein level at 24 h (closed circles) and 48 h (grey circles) were divided by the fold change at the mRNA level as measured by RNA-seq. Black dotted line represents an exact correlation between the fold changes at the protein and mRNA level, and the dashed lines a 4-fold difference between the protein and mRNA levels either up (red) or down (green). See the legend of Fig. 2 for a list of the abbreviations. (TIF) [file ppat.1003938.s005.tif]
