# Supplementary material for: Metabolic Reprogramming during Purine Stress in the Protozoan Pathogen Leishmania donovani
Source: PLoS Pathog. 2014 Feb 27;10(2):e1003938. doi: 10.1371/journal.ppat.1003938 (PMC3937319; doi:10.1371/journal.ppat.1003938)
Supplement: Text S1 — Additional description of materials and methods. (DOCX) [file ppat.1003938.s015.docx]

**SUPPLEMENTARY MATERIALS AND METHODS**

**Comparative Shotgun Proteomics Using Spectral Count Data**

*Sample collection, digestion, chromatographic separations, and mass spectrometry.* Promastigotes of the clonal derivative LdBob from *Leishmania donovani* strain 1S2D were cultured in DME-L with additional supplements, including 100 µM hypoxanthine, as described in the *Materials and Methods*. To induce purine starvation exponentially growing cells were washed twice in DME-L medium with (purine-replete sample) or without 100 µM hypoxanthine (purine-starved samples) and resuspended at 2 x 10^6^ cells ml^-1^ in purine-replete or purine-deplete media, respectively. After 24 h, 2 x 10^8^ cells were harvested from both the purine-replete and purine-starved cultures by centrifugation at 1,500 x *g*, washed once in D-PBS, and resuspended in 0.5 ml of 25 mM ammonium bicarbonate buffer. Cells were disrupted by sonication using a Sonic Dismembrator, model F60 (Fisher Scientific) at a setting of 2 and with 3 x 15 sec bursts with cooling in between. The protein content of each sample was determined by bicinchoninic acid assay using the BCA assay kit (Pierce, Rockville, IL) and 1 mg portions of protein dried by centrifugal evaporation. Samples were suspended in 8M urea buffer, reduced with dithioerythritol, alkylated with idoacetamide, and digested overnight with trypsin. Following addition of formic acid to a final 5% concentration, digests were solid phase extracted on Sep Pak Light Cartridges (Waters Corp., Milford, MA) and peptides separated by strong-cation exchange (**SCX**) chromatography into 32 fractions using a polysulfoethyl A column (PolyLC Inc., Columbia MD). The digestion and SCX methods have been previously described [1]. Forty percent of each cation exchange fraction was then separated by reverse phase chromatography and 100 minutes of tandem mass spectrometry data were collected for each of the 32 fractions using an LTQ linear ion trap (Thermo Scientific, San Jose, CA) operated as described in ref. [2].

*Database searches*

The closest species to *L. donovani* with a sequenced genome at the time of the study was *L. infantum* and RefSeq entries were extracted from a Dec. 28, 2009 download of the NCBI nr database using in-house tools (available at www.ProteomicAnalysisWorkbench.com). A target/decoy database was created by adding 179 contaminant entries to 7,872 *L. infantum* entries and then concatenating the 8,051 sequence-reversed entries. DTA files were created by BioWorks 3.3 (Thermo Scientific) with a molecular weight range of 550 to 4,000, an absolute threshold of 500, 1 group scan, more than 25 ions, and charge state analysis using ZSA. The numbers of DTA files ranged from 405,000 to 466,000 for the four samples. SEQUEST (version 27, rev. 12, Thermo Scientific) searches were configured with an average parent ion mass tolerance of 2.5 Da, a monoisotopic fragment ion tolerance of 1 Da, trypsin cleavage specificity, static +57 Da mass modification of cysteine residues, and variable modifications of +16 Da on methionine and +80 Da on serine, threonine, and tyrosine residues.

*Peptide and protein identification*

A flexible proteomic analysis pipeline developed in-house was used to accurately identify peptides and proteins [3]. Briefly, SEQUEST scores were combined into discriminate function scores [4,5] and histograms of scores for matches to the target and to the decoy database were tabulated for different peptide classes with a 7 amino acid minimum peptide length. The target and decoy score distributions were used to estimate peptide false discovery rates (**FDR**) for each peptide class and set thresholds to filter out incorrect matches. Correct peptide sequences were mapped to protein sequences using peptide-subset-removal parsimony filtering. Peptide sequences from all samples in the experiment were mapped in total followed by removal of any proteins lacking sufficient per sample evidence (two distinct peptides per protein). Peptide thresholds were iteratively determined based on overall protein FDR estimated from decoy protein matches and a peptide FDR of 0.7% was used. Previously, peptides were separated into different classes for FDR analysis based on charge state. In this work, additional classes were added for unmodified peptides, peptides containing M+16 modifications, and peptides containing STY+80 modifications.

*Protein differential expression*

Protein abundances were estimated using spectral counting [6]. Total spectral counts were normalized across samples using quantile normalization, a method that works well in large-scale studies [7]. The counts of peptides mapped to multiple proteins were fractionally split based on the relative unique peptide counts of those proteins. Two methods for determining statistical significance of differential expression candidates were used. A basic Chi square test was computed using the average spectral counts from the purine-replete and purine-deplete samples where the null hypothesis was equal expression in both conditions. A second method ranked proteins from highest total spectral count to lowest, computed the log_2_ of the abundance ratio of the average counts in the two conditions for each protein, and renormalized the ratios (a Z-transfomation using trimmed ratios where the top 5 and bottom 5 ratios were excluded) in a 101-protein sliding window. This removes intensity-dependent dispersion in the expression ratios [8] and is, in essence, an adaptive fold-change test. Candidates from each test were determined by applying a Benjamini-Hochberg correction to p-values [9] and the union of the two sets of candidates was formed. We averaged spectral counts across the 4 samples and imposed a minimum count cutoff of 2.5 to reduce missing data points to negligible levels (less than 1%) and reduce the impact of small count fluctuations.

*Measurement of Resazurin Reduction in Purine-Starved and Purine-Replete Parasites*

Log-phase promastigotes cultured continuously in 100 µM hypoxathine or without purine for 24 h, 48 h, or 2 weeks, were resuspended in growth media plus or minus purine at a density of 5 X 10^8^ cells. A 100 µl of each cell suspension was transferred to a black, polystyrene 96-well plate along with a 10 µl of a 10x resazurin (alamarBlue) solution (Life Technologies). Fluorescence attributable to the reduction of resazurin to resorufin was monitored at 560 nm_ext_ / 590 nm_em_ for 60 min using a SpectraMax M2 Microplate Reader (MolecularDevices GmbH, Ismaning/München, Germany). Heat-killed parasites (heated at 75 ºC for 10 min) and wells without the addition of cells served as controls.

**references**

1. Wilmarth PA, Tanner S, Dasari S, Nagalla SR, Riviere MA, et al. (2006) Age-related changes in human crystallins determined from comparative analysis of post-translational modifications in young and aged lens: does deamidation contribute to crystallin insolubility? J Proteome Res 5: 2554-2566.

2. Bassnett S, Wilmarth PA, David LL (2009) The membrane proteome of the mouse lens fiber cell. Mol Vis 15: 2448-2463.

3. Wilmarth PA, Riviere MA, David LL (2009) Techniques for accurate protein identification in shotgun proteomic studies of human, mouse, bovine, and chicken lenses. J Ocul Biol Dis Infor 2: 223-234.

4. Eng JK, McCormack, A. L., Yates, J. R. (1994) An approach to correlate tandem mass spectral data of peptides with amino acid sequences in a protein database. Journal of the American Society for Mass Spectrometry 5: 976-989.

5. Keller A, Nesvizhskii AI, Kolker E, Aebersold R (2002) Empirical statistical model to estimate the accuracy of peptide identifications made by MS/MS and database search. Anal Chem 74: 5383-5392.

6. Liu H, Sadygov RG, Yates JR, 3rd (2004) A model for random sampling and estimation of relative protein abundance in shotgun proteomics. Anal Chem 76: 4193-4201.

7. Fei SS, Wilmarth PA, Hitzemann RJ, McWeeney SK, Belknap JK, et al. (2011) Protein database and quantitative analysis considerations when integrating genetics and proteomics to compare mouse strains. J Proteome Res 10: 2905-2912.

8. Yang IV, Chen E, Hasseman JP, Liang W, Frank BC, et al. (2002) Within the fold: assessing differential expression measures and reproducibility in microarray assays. Genome Biol 3: research0062.

9. Thissen D, Steinberg L, Kuang D (2002) Quick and Easy Implementation of the Benjamini-Hochberg Procedure for Controlling the False Positive Rate in Multiple Comparisons. Journal of Educational and Behavioral Statistics 27: 77-83.
